# Spatial transcriptomics reveals the heterogeneity and FGG+CRP+ inflammatory cancer-associated fibroblasts replace islets in pancreatic ductal adenocarcinoma

**DOI:** 10.3389/fonc.2023.1112576

**Published:** 2023-04-14

**Authors:** Zhangyong Ren, Bing Pan, Fangfei Wang, Shaocheng Lyu, Jialei Zhai, Xiumei Hu, Zhe Liu, Lixin Li, Ren Lang, Qiang He, Xin Zhao

**Affiliations:** ^1^ Department of Hepatobiliary Surgery, Beijing Chaoyang Hospital affiliated to Capital Medical University, Beijing, China; ^2^ Department of Pathology, Beijing Chaoyang Hospital affiliated to Capital Medical University, Beijing, China

**Keywords:** pancreatic ductal adenocarcinoma, spatial transcriptomics, tumor microenvironment, heterogeneity, cancer-associated fibroblasts

## Abstract

**Background:**

Understanding the spatial heterogeneity of the tumor microenvironment (TME) in pancreatic cancer (PC) remains challenging.

**Methods:**

In this study, we performed spatial transcriptomics (ST) to investigate the gene expression features across one normal pancreatic tissue, PC tissue, adjacent tumor tissue, and tumor stroma. We divided 18,075 spatial spots into 22 clusters with t-distributed stochastic neighbor embedding based on gene expression profiles. The biological functions and signaling pathways involved in each cluster were analyzed with gene set enrichment analysis.

**Results:**

The results revealed that KRT13+FABP5+ malignant cell subpopulation had keratinization characteristics in the tumor tissue. Fibroblasts from adjacent tumor tissue exhibited a tumor-inhibiting role such as “B-cell activation” and “positive regulation of leukocyte activation.” The FGG+CRP+ inflammatory cancer-associated fibroblasts replaced the islets in tumor stroma. During PC progression, the damage to pancreatic structure and function was heavier in the pancreatic exocrine (AMYA2+PRSS1+) than in the endocrine (INS+GCG+).

**Conclusion:**

Our results revealed the spatial heterogeneity of dynamic changes and highlighted the significance of impaired exocrine function in PC.

## Introduction

Pancreatic cancer (PC), also known as pancreatic ductal adenocarcinoma (PDAC), is a devastating malignancy with limited clinical progress. Even after radical surgical resection, the probability of long-term survival of PC patients drops to less than 10%, making PC a global health problem (1). There is still a lack of rational therapeutic approaches in PC treatment. Therefore, improving patient outcomes will depend on deeply clarifying PC heterogeneity, exploring its gene module expression, identifying the markers and functions of tumor stromal cells, and defining the critical elements of cell-cell crosstalk in the tumor microenvironment (TME) (2). Spatial transcriptomics (ST) provides a promising method for elucidating the characteristics and functions of malignant cells in a two-dimensional space, compensating for the lack of spatial mapping of single-cell sequencing.

In short, ST is similar to a high-resolution microscope; it can focus on a compartment of tens of microns, detect and label mRNA, and aid in constructing a gene expression profile at each spot *via* high-throughput sequencing. Currently, ST is used to dissect the heterogeneity of breast cancer *in situ* for genotyping and clinical treatment guidance (3). ST was able to identify tumor-specific keratinocytes in cutaneous squamous carcinoma. Further, it could also reveal the genetic features of cancer-associated fibroblasts (CAFs) in chemo-resistant PC and explore the gene module of inflammatory fibroblasts and cancer cells in PC (4, 5). However, obtaining qualified pancreatic tissue samples remains challenging for TS analysis because of RNA degradation caused by enriched digestive enzymes, tissue ischemic necrosis, and the long duration of surgery. Therefore, there is a lack of ST data for PC, and the spatial distribution and gene expression patterns of various cells in PC remain unclear.

In this study, we generated ST profiles across one normal pancreatic sample (NP) and one PDAC sample, including adjacent tumor tissue (ATT), tumor issue (T), and tumor stroma tissue (TS). We then investigated pancreatic histological heterogeneity to further understand the spatial dynamic changes during PC development.

## Materials and methods

### Specimens and clinical data

From April to October 2022, we harvested one NP tissue from one donor after cardiac death, and T, ATT, and TS tissues from one pathologically confirmed PDAC patient to perform ST. The patients or next of kin provided written informed consent to participate in this study. This study was approved by the Ethics Committee of Beijing Chaoyang Hospital, Capital Medical University (2020-S-274 and 2020-S-302).

We harvested fresh pancreatic tissues from the head of the normal pancreas, tumor stroma tissue from PDAC tumor center, tumor tissue from the pancreatic tumor parenchyma, and adjacent tumor tissue between the tumor margin and the adjacent normal pancreas. The tissues (15 mm square and 5 mm thick) were embedded in OCT and stored at −20°C.

### Spatial transcriptomics

#### Slide preparation

ST slides were printed with four 6.5×6.5 mm capture areas, each with 5,000 spots of barcoded primers (10× Genomics, Pleasanton, CA, USA). Each spot had a diameter of 55 μm and four surrounding spots with a center-to-center distance of 100 μm.

#### Tissue permeabilization

The slides were incubated at 37°C for 20 min with 0.5 U/ml collagenase (Thermo Fisher) and 0.2 mg/ml BSA (NEB, Ipswich, MA) in HBSS buffer (Thermo Fisher). Wells were washed with 0.1 × SSC (Sigma-Aldrich), after which permeabilization was carried out at 37°C for 7 min in 0.1% pepsin (Sigma-Aldrich) dissolved in 0.1 M HCl (Sigma-Aldrich). After incubation, the pepsin solution was removed, and the wells were washed with 0.1 × SSC.

#### Reverse transcription, spatial library preparation, and sequencing

After permeabilization, 70 µL of reverse transcription mix was added to each subarray, ensuring complete coverage of the tissue sections. The hybridization cassette was sealed with a plastic sticker and incubated in a ThermoMixer at 42°C overnight. After reverse transcription, wells were washed with 0.1 × SSC and incubated at 56°C with interval shaking for 1.5 h with a tissue removal mix of Proteinase K (QIAGEN) and PKD buffer (QIAGEN, pH 7.5) at a 1:1 ratio. Supernatants containing the released cDNA were collected and transferred to 96-well plates for ST library. In short, second-strand cDNA synthesis was followed by *in vitro* transcription, adapter ligation, and a second reverse transcription. Sequencing handles and indices were added to an indexing PCR, and the finished libraries were purified and quantified. The libraries were sequenced on the Illumina NextSeq platform with 31 bases from reads 1 and 46 from read 2, and 47–221 million raw reads were generated per sample.

#### Spot visualization and image alignment

Primer spots were stained by hybridization of fluorescently labeled probes and imaged on a Metafer Slide Scanning platform. The resulting spot image was loaded into the web-based ST Spot Detector tool, along with the previously obtained BF tissue image of the same area. The two images were aligned, and a built-in tissue recognition tool was used to extract spots covered by the tissue.

#### Spatial transcriptomics raw data processing

Raw sequencing data were processed using the open-source ST pipeline v1.7.2 with the GRCh38 v86 genome assembly as a reference and the corresponding GENCODE annotation file (version 25). The ST pipeline was executed with a two-pass mode that enabled alignment, and reads in which the UMI had at least six low-quality bases were discarded. The count matrixes were filtered to retain only protein-coding, long non-coding, and antisense genes.

#### Spatial transcriptomics data processing

The gene-spot matrixes from the ST and Visium samples were analyzed using the Seurat R package (versions 3.0.0/3.1.3). For each data sample, spots were filtered for a minimum detected gene count of 200 genes, whereas genes with fewer than 10 read counts or those expressed in fewer than two spots were removed. Normalization across spots was performed using the SCTransform function with regression of replicates and the number of genes per spot.

Dimensionality reduction and clustering were performed using independent component analysis at a resolution of 0.8 with the first 20 ICs. For merged data, all replicate samples were merged with the ‘‘merge’’ Seurat function and renormalized with SCTransform prior to independent component analysis and t-distributed stochastic neighbor embedding (t-SNE) on the first 20 ICs. The correlation matrix of the spatial cluster genes was generated by first taking differentially expressed genes (average log fold changed (logFC) > 0.25, adjusted p-value < 0.05, by Wilcoxon Rank Sum test) across ST clusters. Pearson’s correlation was calculated across this matrix, and hierarchical clustering of ST clusters was performed using the heatmap function in the gplots R package.

Signature scoring derived from ST signatures was performed using the AddModuleScore function with the default parameters in Seurat. Nearest neighbor analysis was performed by counting the cluster identities of replicate sections. A null distribution of neighboring spots was determined by shuffling the cluster identities and counting the nearest neighbor identities across randomized data for 1,000 permutations while preserving the number of spots and cluster identities per tissue section. The p-value was the number of randomized permutations that exceeded the observed data. Spatial feature expression plots were generated using the SpatialFeaturePlot function in Seurat (version 3.1.3) and the STUtility R package (version 1.0.0). A Loupe browser (version 6.0) was used to generate ST figures.

### Biological informatics analysis

Firstly, gene expression values were normalized, and the genes with low expression levels were omitted. To explore the biological function of a cluster of spots, we compared the expression value of this cluster with the average value of all the clusters within the same sample. We then obtained the genetic FC and ranked the gene list according to the log_2_FC. We used the clusterProfiler R package to complete gene set enrichment analysis, and the results were visualized using the ggplot2 R package. Canonical pathways were used as the background gene set for the pathway enrichment analysis. Biological process (BP) analysis was performed using Gene Ontology gene sets. We used the DisGeNET database to predict the diseases linked to the specific gene patterns of the cluster (6). In addition, we used the PaGenBase and Cell Type Signature databases to identify the tissue and cellular specificity of cluster gene sets, respectively (7, 8). Protein–protein interaction networks were constructed using the Metascape network tool and hub genes were identified using the Molecular Complex Detection approach (9).

### Multiple immunofluorescence

The PC tissue was embedded in paraffin and then cut into 5 μm-thick pathological sections. Immunofluorescence staining was performed using the following primary antibodies: THBS1 (1/5000, Abcam, ab267388), CK7 (1/8000, Abcam, ab181598), and α-SMA (1/100, Abcam, ab32018). We then used a multi-color immunofluorescence kit (Opal™ 4-Color Manual IHC Kit, PerkinElmer) and followed the manufacturer’s instructions to visualize the above molecular indicators. Immunofluorescence images were captured using an Olympus microscope (Olympus BX43).

### Immunohistochemistry

In brief, we sectioned the paraffin-embedded tissue specimens and incubated them with anti-THBS1, -CRP, and -FGG primary antibody (1 : 5000, Abcam, ab267388) overnight at 4°C. The slides were incubated with an HRP-conjugated secondary antibody (goat anti-rabbit IgG; BOSTER, Hangzhou, China) for 1 h. We used a 3,3′-diaminobenzidine substrate solution as a chromogen to visualize THBS1, CRP, and FGG.

### Statistical analysis

The limma R package was used to analyze the differential expression genes. Pearson’s correlation test was applied for correlation analysis. A value of *p* < 0.05 was considered statistically significant.

## Results

### Identification of diverse pancreatic tissues with marker genes

**Figure 1:**
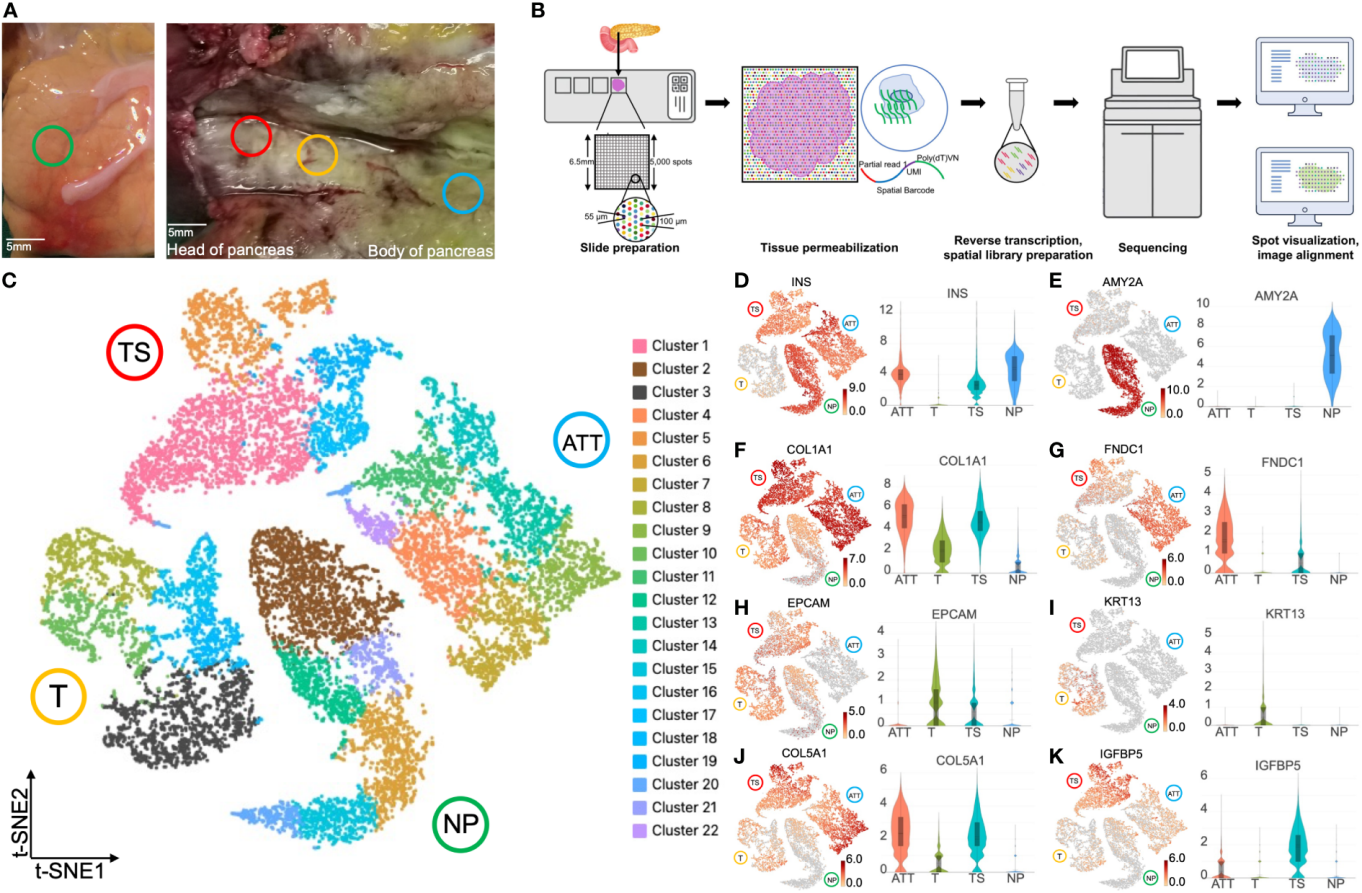
Spatial transcriptomics clustering and marker genes of four pancreatic tissues at different locations. **(A)** Normal pancreas tissue (NP, green circle) and pancreatic ductal adenocarcinoma tissues, including tumor (T, yellow circle), tumor stroma (TS, red circle), and adjacent tumor tissue (ATT, blue circle), were selected to perform spatial transcriptomics. **(B)** The experimental flow chart of this study. **(C)** t-SNE plot of 18,075 spots from the four pancreatic tissues showing 22 clusters in different colors. Cluster 3 (black spots) overexpressed KRT13 and was identified as malignant cells. Cluster 2 (brown spots) highly expressed AMY2A and EPCAM, and was determined to be healthy pancreatic ductal cells. Gene expression intensity distribution plots and violin plots of INS **(D)**, AMY2A **(E)**, COL1A1 **(F)**, FNDC1 **(G)**, EPCAM **(H)**, KRT13 **(I)**, COL5A1 **(J)**, and IGFBP5 **(K)**. The bar plots and the vertical coordinate of the violin plots indicates the log_2_FC of the gene expression value of each cluster versus the average gene value.

We chose NP, ATT, T, and TS to perform tissue sectioning and hematoxylin and eosin (H&E) staining (**Figure 1A**). The experimental procedure was shown in **Figure 1B**. The median genes detected by each spot ranged from 853 to 2,610 in the four sections (**Figure S1**). These spots could be grouped into 22 clusters based on t-SNE (**Figure 1C**). To test the efficiency of ST in identifying different tissues, we determined the expression levels of the marker genes in these tissues. NP, ATT, and TS expressed INS (insulin); however, T did not express INS because of the lack of islets (**Figure 1D**). AMY2A (amylase) was distinctively expressed in NP (**Figure 1E**) but not in T, ATT, and TS. This highlighted the replacement of the original exocrine cells by malignant or stromal cells. In line with PC tissue fibrosis, COL1A1 (collagen type I alpha 1 chain, a fibroblast marker gene) levels were higher in ATT, TS, and T than in NP (**Figure 1F**). As a fibroblast marker (10), FNDC1 (fibronectin type III domain containing 1) was expressed most prominently in TS (**Figure 1G**). EPCAM (epithelial cell adhesion molecule), a feature gene of PDAC (11), was highly expressed in T and TS (**Figure 1H**), and KRT13 [keratin 13 and an epithelial-derived antigen (12)] was specifically expressed in T (**Figure 1I**). Additionally, the expression of COL5A1 [collagen type V alpha 1 chain and a fibroblasts hallmark gene (13)] was upregulated in cancer tissues compared to that in NP and ATT (**Figure 1J**); however, IGFBP5 (insulin like growth factor binding protein 5), a protein secreted by CAFs (14), was highly expressed in TS (**Figure 1K**). Taken together, the differences in marker genes exhibited by ST were consistent with our understanding of the gene expression characteristics in the four pancreatic tissues.

### Malignant cells showed keratinization features

Using ST, we can precisely identify cancer cell and normal pancreatic ductal cell populations based on the expression of marker genes. As KRT13 was specifically expressed in tumor tissues, we selected the cluster 3 (including 1,506 spots) with the highest KRT13 log_2_FC in malignant cells (**Figure 1C**). Parallelly, the cluster 2 (including 1,925 spots) was determined to be healthy pancreatic duct cells with the highest abundance of AMY2A (**Figure 1C**). Next, we identified the differentially expressed genes in these two groups and sorted them according to log_2_FC. We then performed gene set enrichment analysis (GSEA) based on the canonical pathways gene set to query functional changes in cancer cells. The results showed that the upregulated genes were substantially enriched in the keratinization and glycolysis pathways (**Figure S2A**). In contrast, downregulated genes were associated with the matrix metalloproteinase and EGFR tyrosine inhibitor resistance (**Figure S2B**).

### Localization of endocrine and exocrine parts in normal pancreas

**Figure 2:**
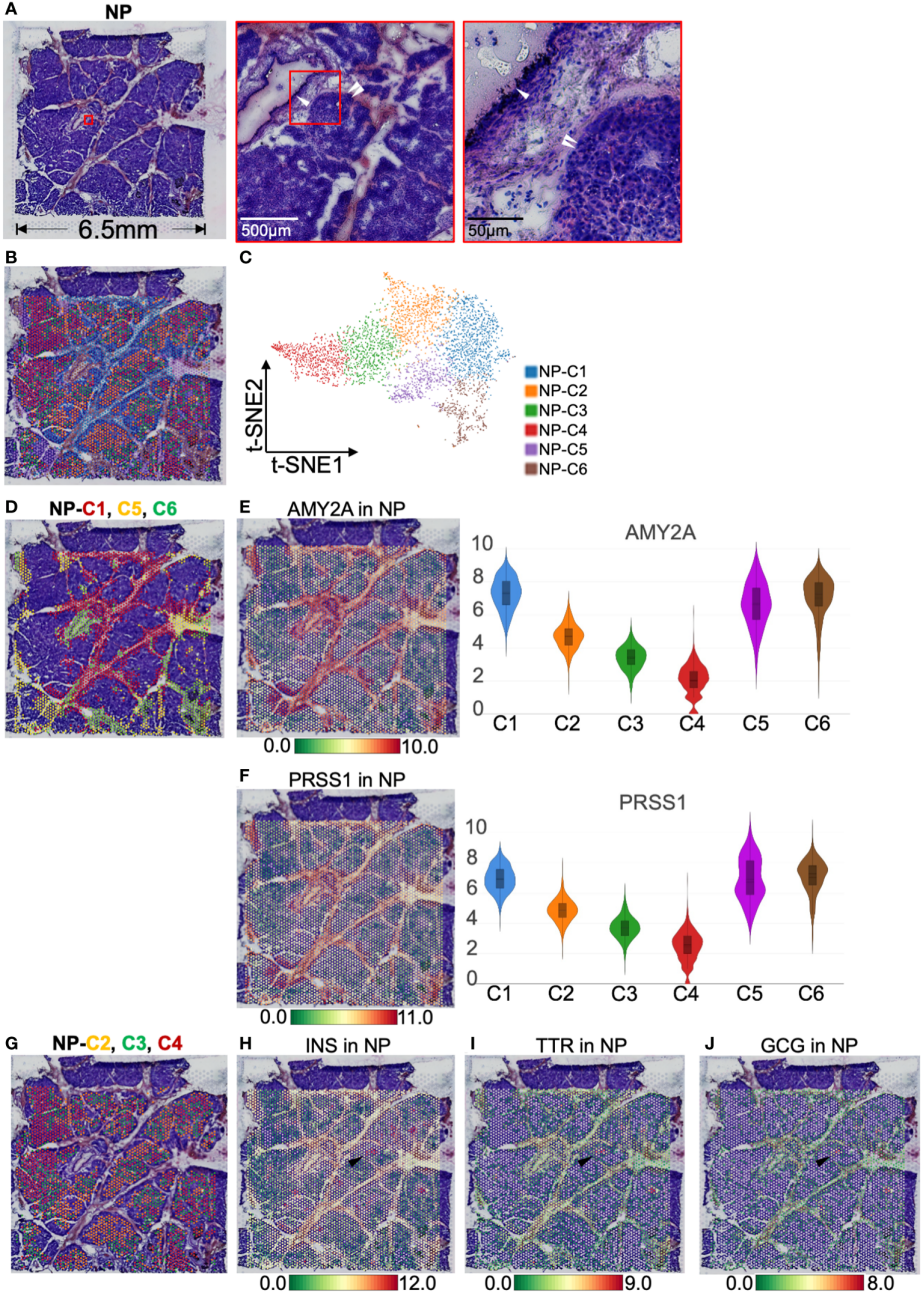
Clustering and spatial localization of endocrine and exocrine parts in normal pancreas (NP). **(A)** Hematoxylin and eosin (H&E) images of NP. The red box shows pancreatic duct (white single arrow) and islets (white double arrow). **(B)** A total of 4,724 spots were grouped into six clusters in different colors and were distributed in the NP compartment. **(C)** t-SNE plot of spatial transcriptomics indicating six clusters. **(D)** H&E image showing NP-Cluster (C)1 (red spots), -C5 (yellow spots), and -C6 (green spots) are located around pancreatic ducts. Spatial distribution and expression abundance of AMY2A **(E)** and PRSS1 **(F)**. **(G)** H&E image showing NP-C2 (red spots), -C3 (green spots), and -C4 (red spots) occupy most of the NP area, surrounded by pancreatic ducts. Spatial distribution and expression level of INS **(H)**, TTR **(I)**, and GCG **(J)**. Black arrows highlight the spots at the same position and simultaneously express INS, TTR, and GCG. The bar plots and the vertical coordinate of the violin plots indicates the log_2_FC of the gene expression value of each cluster versus the average gene value.

We first observed the gene expression and distribution in the NP with ST. H&E images showed that islets were segmented by interleaved pancreatic ducts, and 4,724 spots covered this area (**Figures 2A, B**). These spots were divided into six clusters according to t-SNE (**Figure 2C**). The top 10 genes defining each cluster are listed in **Table S1** and visualized using a heatmap (**Figure S3A**). NP-cluster(C)1, -C5, and -C6 located around the pancreatic duct and expressed high levels of AMY2A and PRSS1 (trypsinogen, **Figures 2D–F**). Therefore we determined these three clusters as exocrine cells. In contrast, NP-C2, -C3, and -C4 were scattered and surrounded by pancreatic ducts, expressing INS, GCG (glucagon), and TTR (transthyretin, an islet β-cell functional protein), thus marking the location of islets (**Figures 2G–J**). Consequently, NP-C2, -C3, and -C4 were identified as endocrine cells.

### Spatial transcriptomics revealed islet fibrosis and tumor-inhibiting fibroblasts in adjacent tumor tissue

**Figure 3:**
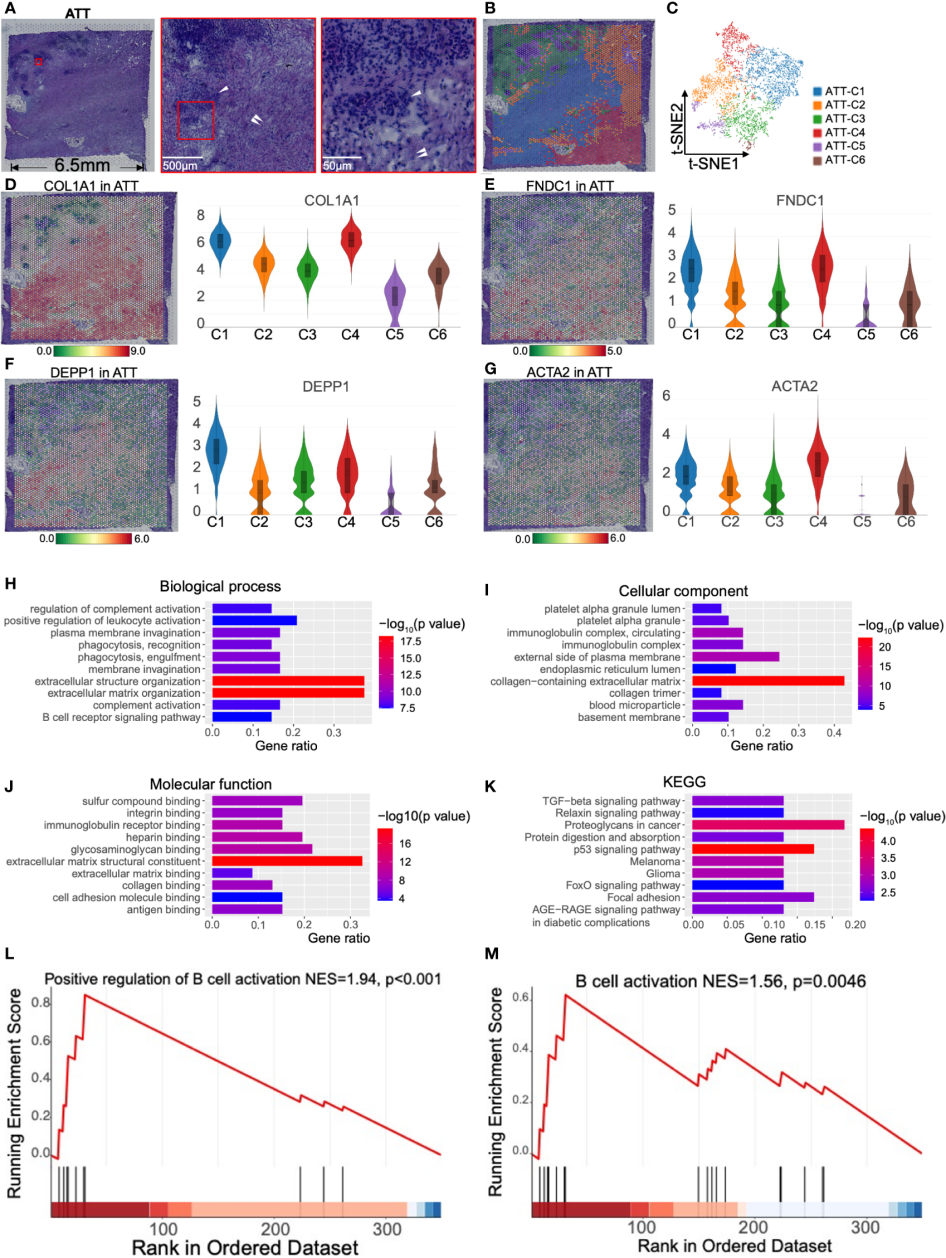
Clustering and spatial distribution of the spots from adjacent tumor tissue (ATT). **(A)** Hematoxylin and eosin stained images of ATT. The red box shows islets (white single arrow) and stromal tissue (white double arrow). The lower right is adjacent to the tumor, and the upper left is near the normal pancreas. **(B)** The spots were grouped into six clusters and distributed to the ATT compartment. **(C)** t-SNE plot of the six clusters. The level and distribution of COL1A1 **(D)**, FNDC1 **(E)**, DEPP1 **(F)**, and ACTA2 **(G)** in ATT. Gene Ontology enrichment analysis of the top 50 genes of ATT-C4, including biological process **(H)**, cellular component **(I)**, and molecular function **(J)**. **(K)** Kyoto Encyclopedia of Gene and Genomes (KEGG) pathway enrichment analysis of ATT-C4. **(L, M)** Gene set enrichment analysis shows that ATT-C4 genes are significantly associated with B cell activation. The bar plots and the vertical coordinate of the violin plots indicates the log_2_FC of the gene expression value of each cluster versus the average gene value.

To understand the effect of PDAC on normal pancreatic tissues, we selected the adjacent tumor tissue for spatial transcriptomics. In pathological sampling, we defined the direction and location of the sections; i.e., the lower right part of the H&E stained section was close to the tumor, and the upper left part was close to the normal pancreas (**Figure 3A**). The H&E image showed islets and abundant stromal tissue (**Figure 3A**). According to the K-means clustering algorithm, we divided the 4,885 spots into six clusters (**Figures 3B, C**). The top 10 genes defining each cluster are shown in **Figure S3B** and **Table S2**. ATT-C1 and -C4 occupied a major portion of the pathological section space and expressed high levels of fibroblast markers such as COL1A1 (15) and FNDC1 (16) (**Figures 3D, E**) and a low level of KRT13 (**Figure 1I**). Therefore, these two clusters were identified as fibroblasts. In addition, ATT-C1 specifically overexpressed DEPP1 (DEPP autophagy regulator 1, **Figure 3F**), which can activate MAPK signaling and is related to pancreatic stromal sclerosis (17). BP analysis further revealed that ATT-C1 genes were related to extracellular matrix (ECM) remodeling, such as “core matrisome” and “ECM glycoproteins” (**Figure S4**).

ATT-C4 highly expressed ACTA2, which is a conventional fibroblast marker encoding α-SMA (**Figure 3G**). We performed Gene Ontology analysis based on the top 50 genes of ATT-C4, and this gene set was enriched in “positive regulation of leukocyte activation” and “B cell receptor signaling pathway” in BP (**Figure 3H**). The gene set of ATT-C4 was consistently enriched in “immunoglobulin complex” and “immunoglobulin receptor binding” in cellular component and molecular function, respectively (**Figures 3I, J**). Kyoto Encyclopedia of Gene and Genomes analysis showed that ATT-C4 top genes were involved in “proteoglycans in cancer” and “p53 signaling pathway” (**Figure 3K**). Further, GSEA revealed that the gene functions of ATT-C4 were associated with “positive regulation of B cell activation” and “B cell activation” (**Figures 3L, M**). In addition, ATT-C4 is also linked to “extracellular matrix organization” and “regulation of complement activation” (**Figure 3H**). Thus, our results suggest that the fibroblasts from adjacent tumor tissue exhibit tumor-promoting effects and construct a tumor-limiting state.

**Figure 4:**
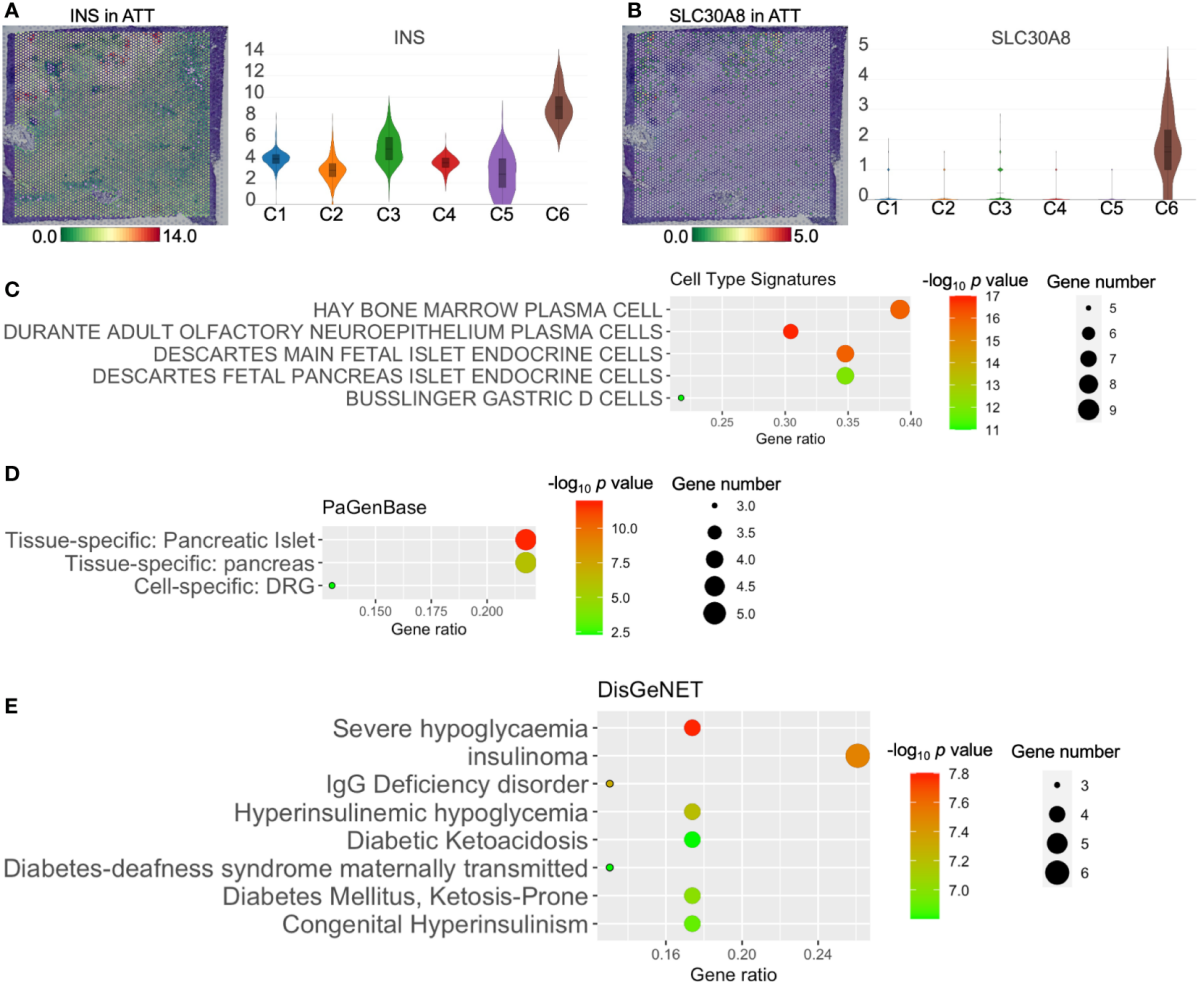
The functions of adjacent tumor tissue (ATT)-cluster(C)3, -C5, and -C6. The distribution and level of INS **(A)** and SLC30A8 **(B)** in ATT. Cell type signature **(C)**, PaGenBase **(D)**, and DisGeNet **(E)** analysis of ATT-C3 upregulated genes. The bar plots and the vertical coordinate of the violin plots indicates the log_2_FC of the gene expression value of each cluster versus the average gene value.

H&E staining suggested clusters 5 and 6 coincided with the location of islets (**Figures 3A, B**). ATT-C6 was determined to be a functional islets for expressing INS and SLC30A8 (solute carrier family 30 member 8, a protein specifically expressed in the islets of Langerhans, **Figures 4A, B**). However, both genes were expressed at low levels in ATT-C5 (**Figures 4A, B**). Interestingly, ATT-C3 encircled the islet regions but was distinct from the islets in the H&E image (**Figures 3A, B**). Instead, ATT-C3 resembled the staining of fibroblast areas (ATT-C1 and -C4) and showed lower expression of COL1A1 and FNDC1 (**Figures 3D, E**). Furthermore, “Cell Type Signature” analysis revealed that ATT-C3 had the characteristics of islet endocrine cells (**Figure 4C**). “PaGenBase” analysis also indicated that this cluster was significantly related to the tissue-specific pancreatic islets (**Figure 4D**). “DisGeNet” analysis suggested that this cluster was associated with insulinoma (**Figure 4E**). Collectively, these results indicated that ATT-C3 presumably represents the impaired islets.

### Spatial transcriptomics recognized the heterogeneity of pancreatic cancer tissue

**Figure 5:**
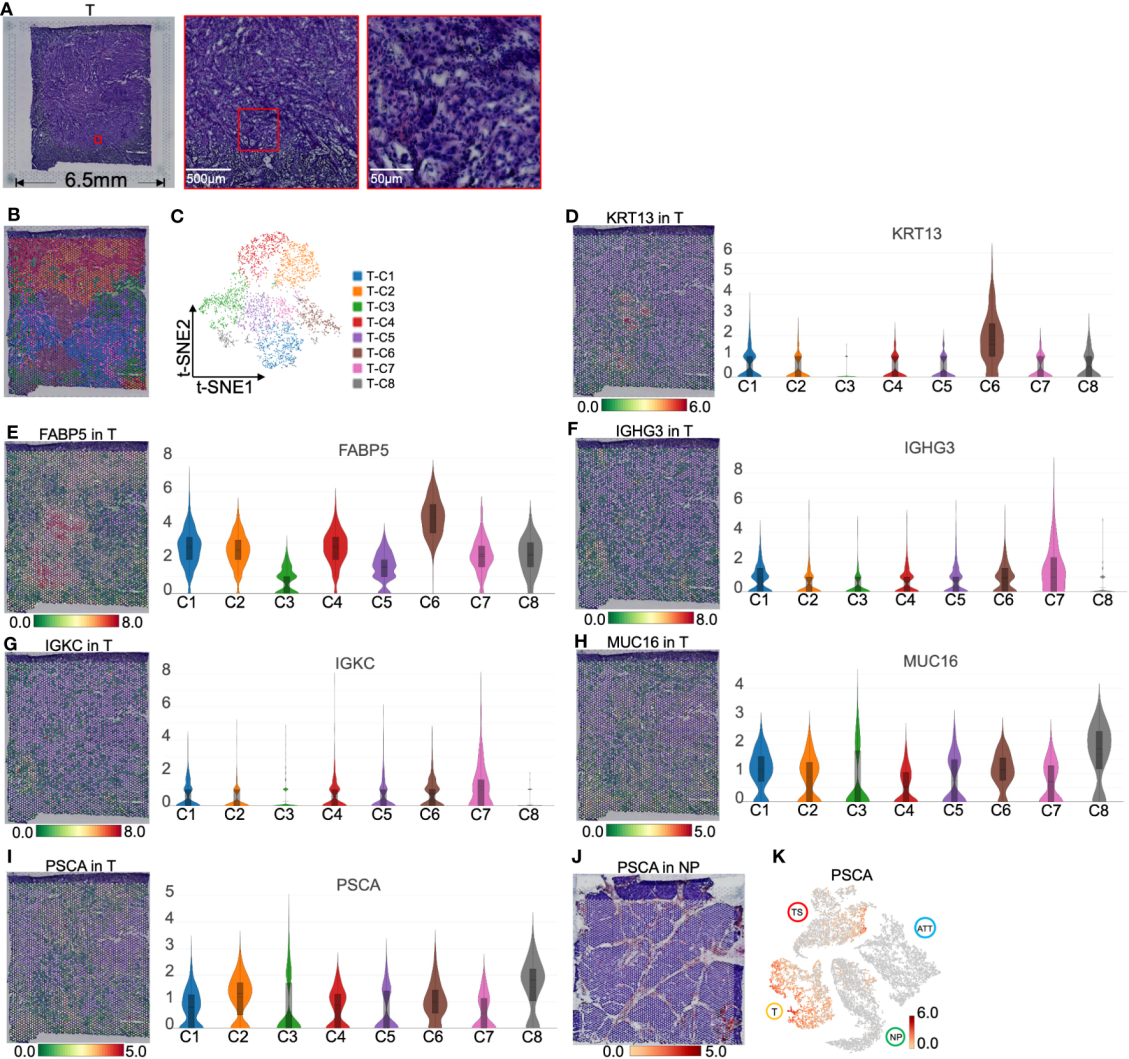
Clustering and spatial distribution of the spots from pancreatic tumor tissue (T). **(A)** Hematoxylin and eosin images of tumor section. **(B)** Spatial distribution of eight clusters in tumor tissue. **(C)** t-SNE plot of the eight clusters of tumor tissue. The level and distribution of KRT13 **(D)**, FABP5 **(E)**, IGHG3 **(F)**, and IGKC **(G)**, MUC16 **(H)** and PSCA **(I)**. **(J)** PSCA location in normal pancreas. **(K)** Distribution of PSCA across normal pancreas (NP), adjacent tumor tissue (ATT), tumor tissue (T), and tumor stroma (TS). The bar plots and the vertical coordinate of the violin plots indicates the log_2_FC of the gene expression value of each cluster versus the average gene value.

H&E staining of PC tumor tissue revealed dense nests of tumor cells lacking stromal components (**Figure 5A**). To illustrate PC heterogeneity, we performed ST on 3,983 spots of this tumor section and divided these spots into eight clusters according to t-SNE. (**Figures 5B, C**). The top 10 genes of each cluster are shown in **Figure S3C** and **Table S3**. We found that T-C6 was relatively clustered, whereas the other groups were more scattered (**Figure 5B**). Spatial transcriptomics indicated that T-C1 was dispersed and relatively overexpressed in LAMC2 (laminin subunit gamma 2, an extracellular matrix glycoprotein) and CEACAM6 (CEA cell adhesion molecule 6, **Figures S5A–C**). BP analysis showed that the gene functions of T-C1 were related to “NABA matrisome-associated” and “NABA secreted factors,” which suggested that T-C1 plays a role in the mesenchymal transition and ECM formation (**Figure S5D**). T-C2 comprised one-fourth of the tumor section and showed relatively high expression of TNNI2 (troponin I2, a fast-twitch skeletal muscle protein) and SYT8 (synaptotagmin 8 and a membrane protein that is important in neurotransmission and hormone secretion, **Figures S5E–G**), which have been found to promote PC (18, 19). The GSEA results suggested that the gene function of T-C2 was closely related to “the positive regulation of immune response” (**Figure S5H**). We did not screen for T-C5 marker genes. However, regarding BP terms, the upregulated genes of T-C5 were enriched in “cytoplasmic translation” and immune-activating functions (**Figure S5I**).

T-C6 was concentrated in a small area in the tumor section, with the overexpression of KRT13 and FABP5 (encoding a fatty acid binding protein found in epidermal cells), which suggested that these spots primarily represented the tumor cells derived from the pancreatic ductal epithelium (**Figures 5D, E**). We also found overexpression of APOL1 (apolipoprotein L1, a secreted high density lipoprotein) and RHCG (Rh family C glycoprotein) in this cluster (**Figure S6**). APOL1 has been reported to define a ductal subpopulation in PDAC using ST and promote PC progression (20). Although the role of RHCG in PC is unknown, it has been identified as a tumor-type marker in renal cancer (21). Due to the low levels of collagen and immunoglobulin genes in T-C6, we hypothesized that this group was dominated by malignant cells. T-C7 was scattered around T-C6 and was relatively high in immunoglobulins such as IGHG3 (immunoglobulin heavy constant gamma 3 involved in B-cell activation) and IGKC (immunoglobulin kappa constant, **Figures 5F, G**), which are markers secreted by tumor-infiltrating B cells (22, 23). This is most likely because T-C7 is a mixture of cancer cells and infiltrating immune cells.

Interestingly, T-C8 was found at the edge of the tumor slide (**Figure 5B**), with high levels of MUC16 (a O-glycosylated protein of mucins) and PSCA (a prostate stem cell antigen and a specific marker of prostate cancer, **Figures 5H, I**). Mucins are found on the apical surfaces of epithelial cells and form a barrier to protect epithelial cells from pathogens. Thus, it has been revealed that MUC16 can be used as a PC marker and is significantly associated with the poor prognosis of PC (24). We further investigated that PSCA primarily located near pancreatic ducts was upregulated in tumor tissues (**Figures 5J, K**). These results suggested that PSCA is expressed in epithelial cells, especially tumor cells.

### FGG+CRP+ inflammatory cancer-associated fibroblasts replaced islets in pancreatic cancer stroma

**Figure 6:**
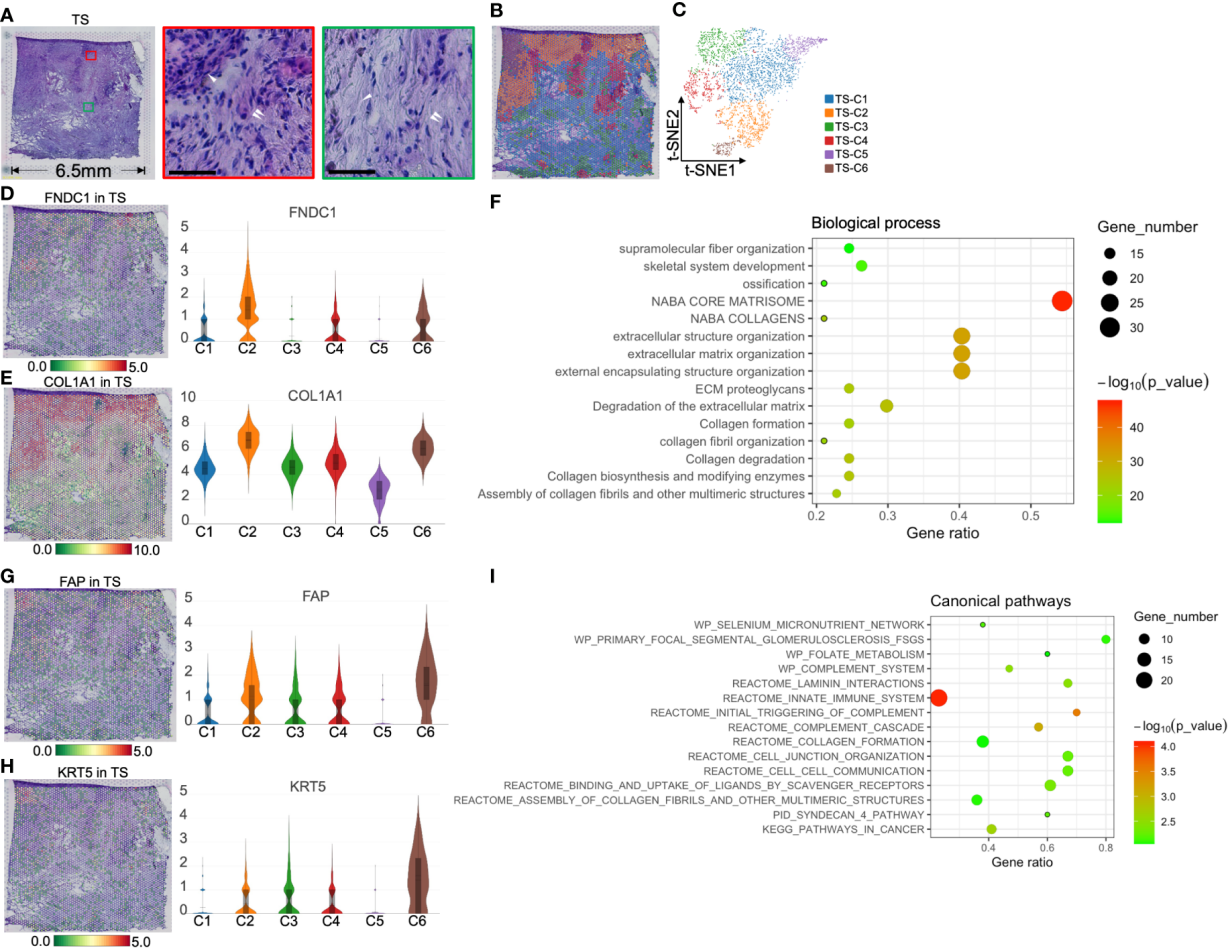
Clustering and spatial distribution of the spots from tumor stroma (TS). **(A)** Hematoxylin and eosin stained images of the tumor stroma. The red box and the middle image show islet cells (white single arrow) and the surrounding extracellular matrix and CAFs (white double arrow). The green box marks the necrotic area (white single arrow), showing the rich extracellular matrix and a few stromal cells (white double arrow). Scale bars = 50 μm. Spatial distribution **(B)** and t-SNE plot **(C)** of six clusters in tumor stroma. The level and distribution of FNDC1 **(D)** and COL1A1 **(E)** in tumor stroma. **(F)** Biological process analysis for TS-Cluster (C)2. The level and distribution of FAP **(G)** and KRT5 **(H)** in tumor stroma. **(I)** The canonical pathway enrichment analysis for TS-C6. The bar plots and the vertical coordinate of the violin plots indicates the log_2_FC of the gene expression value of each cluster versus the average gene value.

ECM can play a critical regulatory role in tumor metastasis and drug resistance; we aimed to dissect the spatial organization of tumor stroma. Notably, H&E staining of the tumor stromal section revealed steatosis and necrosis close to the tumor center; however, islets (the large dark purple areas) were far from the center (**Figure 6A**). The gene module expression of ST divided the entire region of 4,483 spots into six groups (**Figures 6B, C**). The top 10 marker genes of each cluster are shown in **Figure S3D** and **Table S4**.

Because TS-C2, -C4, and -C6 are intrinsically related in function and location, we first discuss these three cell groups. TS-C2 was located between cancer cells and islets and expressed FNDC1 and COL1A1 (**Figures 6D, E**). BP analysis showed that TS-C2 top 50 genes were enriched in ECM reconstruction, including “the degradation of extracellular matrix” and “ECM proteoglycans” (**Figure 6F**). Subsequent protein–protein interaction networks demonstrated that COL1A1, COL6A2, and COL2A1 were the core proteins of TS-C2 (**Figure S7**); this cluster was predicted to be myofibroblastic CAFs (myCAFs).

Combined with H&E staining and ST, we found that TS-C6 was circled by myCAFs (TS-C2, **Figure 6B**) and expressed FAP [fibroblast activation protein alpha, a marker of CAFs (25)] and KRT5 (keratin 5, **Figures 6G, H**), which provided evidence that this cluster is a mixture of tumor cells and CAFs. Subsequently, canonical pathway enrichment of GSEA also supported that the gene functions of this cluster were linked to CAFs and tumor cells, such as “assembly of collagen fibrils,” “collagen formation,” and “pathways in cancer” (**Figure 6I**).

**Figure 7:**
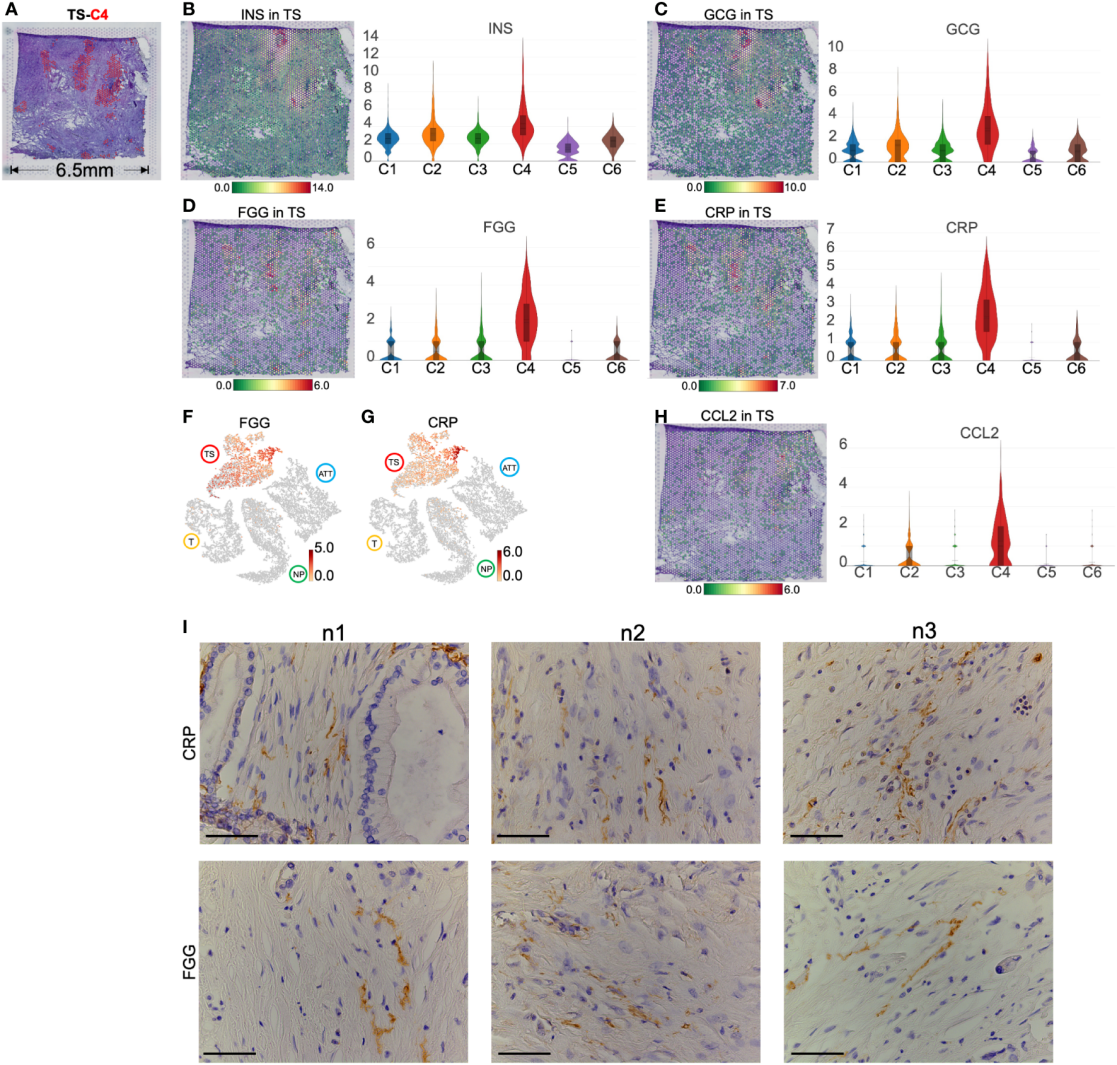
Marker genes and spatial distribution of tumor stroma (TS) -cluster (C)4. **(A)** Distribution of TS-C4 (the red spots) in hematoxylin and eosin images image. The level and distribution of INS **(B)**, GCG **(C)**, FGG **(D)**, and CRP **(E)** in TS. **(F, G)** The distribution and level of FGG and CRP across normal pancreas (NP), adjacent tumor tissue (ATT), tumor (T), and TS. **(H)** The level and distribution of CCL2 in TS. **(I)** Immunohistochemical staining of FGG and CRP in the TS from three pancreatic ductal adenocarcinoma tissue samples. The bar plots and the vertical coordinate of the violin plots indicates the log_2_FC of the gene expression value of each cluster versus the average gene value. Scale bar = 50 μm.

H&E staining showed that the distribution of TS-C4 coincided with the location of the islets (**Figure 7A**). Simultaneously, a small number of spots expressed INS and GCG, indicating that INS+GCG+ spots represent functional islets (**Figures 7B, C**). In contrast, the other regions did not express INS and GCG but highly expressed FGG (fibrinogen gamma chain, a fibroblast marker (26), **Figure 7D**) and CRP (C-reactive protein and a classic inflammatory factor (27), **Figure 7E**). Importantly, both genes were specifically expressed in the islet region of the TS (**Figures 7F, G**). Moreover, TS-C4 also specifically overexpressed CCL2 (**Figure 7H**), a cytokine secreted by CAFs in PC (28, 29). We performed immunohistochemical staining on the adjacent sections, and found that the tumor stroma was rich in cells that were arranged in sheets like flowing water with elliptical nuclei, which is a typical feature of CAFs. Cord-like distribution of FGG and CRP protein staining was observed in the middle of these cells, suggesting the presence of FGG+CRP+ CAFs (**Figure 7I**).

TS-C1 occupied the largest area of the tumor slice, and TS-C5 was located in the necrotic area inside TS-C1 (**Figure 6B**). Unfortunately, no typical genes or pathological differences were observed between the two clusters. The wiki pathway enrichment of GSEA showed a similar “oxidative phosphorylation” function for the two groups (**Figures S8A, B**). Additionally, TS-C3 encircled the islet region and displayed multiple functions, such as vasculature development, immune response, and collagen organization. (**Figure S8C**).

### Spatial transcriptomics identified that THBS1 was primarily expressed in the tumor stroma

**Figure 8:**
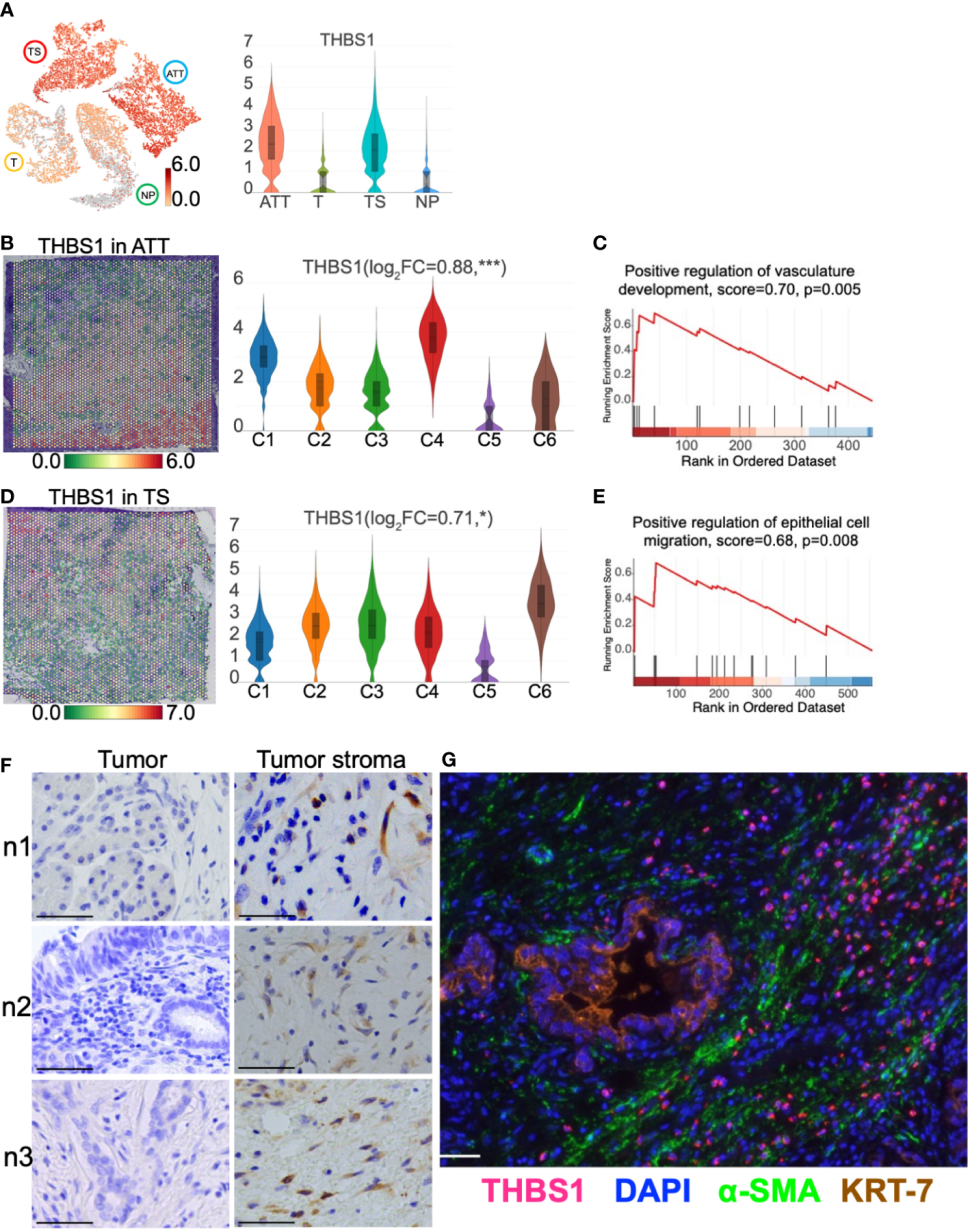
THBS1 location and distribution in pancreatic tissues. **(A)** The distribution and level of THBS1 across the normal pancreas (NP), adjacent tumor tissue (ATT), tumor (T), and tumor stroma (TS). **(B)** The distribution and level of THBS1 in ATT. **(C)** ATT-C4 genes were enriched in “Positive regulation of vasculature development” by gene set enrichment analysis (GSEA). **(D)** The distribution and level of THBS1 in TS. **(E)** TS-C6 genes were enriched in “Positive regulation of epithelial cell migration” by GSEA. **(F)** Immunohistochemical staining of THBS1 in the tumor (left column) and tumor stromal tissues (right column) from three pancreatic ductal adenocarcinoma tissue samples. **(G)** Multiple immunofluorescence staining in tumor tissue. The bar plots and the vertical coordinate of the violin plots indicates the log_2_FC of the gene expression value of each cluster versus the average gene value. Scale bar = 50 μm. *Represents a statistical P-value<0.05, and *** represents a statistical P-value<0.001.

Accumulated evidence indicates that PC stromal cells, especially CAFs, can construct an immunosuppressive TME and promote tumor cell invasion and metastasis. Thus, upregulated genes in ATT and TS were screened out to obtain key genes. As a result, thrombospondin1 (THBS1) expression was upregulated in TS and ATT, as determined *via* ST detection (**Figure 8A**). Specifically, THBS1 was highly expressed in ATT-C4, which is closely related to vascular development (**Figures 8B, C**). Moreover, THBS1 levels increased in TS-C6, which is involved in promoting epithelial cell migration (**Figures 8D, E**). Using multiple immunofluorescence and immunohistochemistry, we then validated that THBS1 was primarily expressed in tumor stroma but not in tumor cells in the same sample (**Figures 8F, G**). Consequently, we provided evidence that THBS1 was expressed in the TS, despite the presence of tumor tissue, which was consistent with the results of ST.

### Spatial transcriptomics revealed the variation of CD81 between normal and cancerous pancreases

**Figure 9:**
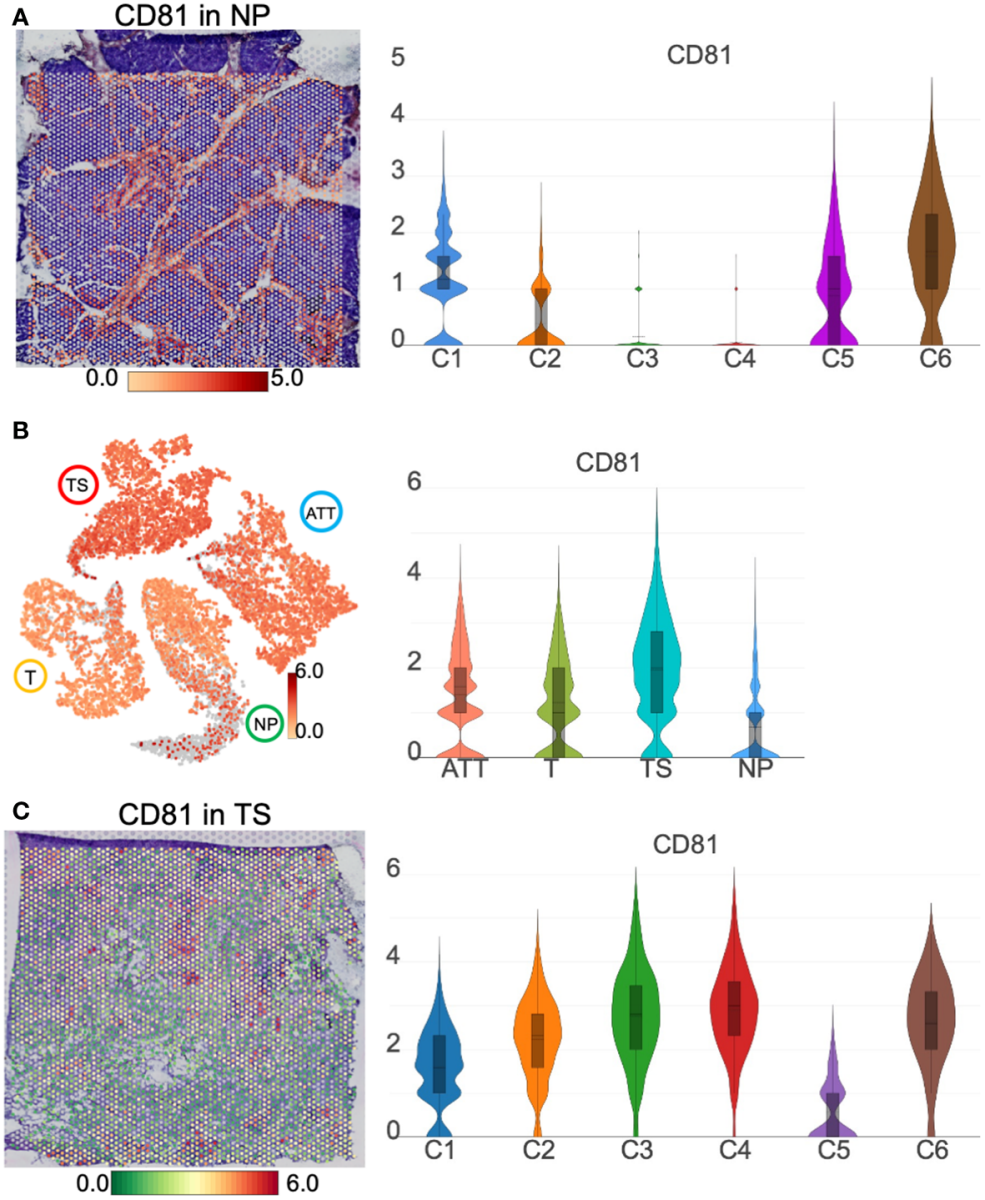
CD81 location and distribution in pancreatic tissues. **(A)** The distribution and level of CD81 in normal pancreas (NP). **(B)** The distribution and level of CD81 across the NP, adjacent tumor tissue (ATT), tumor (T), and tumor stroma (TS). **(C)** The distribution and level of CD81 in TS. The bar plots and the vertical coordinate of the violin plots indicates the log_2_FC of the gene expression value of each cluster versus the average gene value.

As an exosomal marker protein, CD-81 (a member of the transmembrane 4 superfamily) is expressed in PC cell lines and pancreatic fluid exosomes and is expected to be used as a molecular marker for liquid biopsy. Here, we demonstrated that CD81 was clearly located in the pancreatic duct region and barely expressed in islets from the NP (**Figure 9A**). As islet cells accounted for majority of the normal pancreatic section, the CD81 average expression was the lowest in the NP among the four pancreatic tissues. For cancer tissues, the number of ductal epithelial cells increased; thus, the CD81 mean level was higher in cancer tissues than that of the NP (**Figure 9B**). CD81 expression was highest in the TS, especially in the iCAFs subgroup (TS-C4) that replaced islets (**Figure 9C**), indicating that CD81 was primarily produced by iCAFs.

## Discussion

Previously, we used single-cell RNA sequencing technology to determine the biological functions of diverse cell subgroups in PC (30). We further analyzed the substantial spatial heterogeneity of PC. Marker genes of normal pancreatic endocrine and exocrine cells showed distinct spatial distribution features. Previous studies have shown that AMY2A and PRSS1 are overexpressed in pancreatic acinar cells (5). In the current study, we found that AMY2A and PRSS1 are only presented around the pancreatic duct with a uniform distribution, suggesting that acinar cells are located adjacent to the pancreatic ducts. In contrast, insulin and glucagon are specifically and overlappingly expressed in a small number of islets; the islets that produce insulin also secrete glucagon. Interestingly, compared to that in NP, amylase was not expressed in tissues of T, ATT, and TS. Meanwhile, the insulin level was decreased in ATT and TS and was not observed in T. This suggests that during PC occurrence and progression, the pancreatic exocrine part is the first to be destroyed entirely; although the endocrine part is also impaired, it still retains some functions. In clinical practice, new-onset diabetes is considered to be a high-risk factor for PC (31). Our results suggest that changes in exocrine function caused by PC are more sensitive than changes in endocrine function. This new finding will encourage the search for molecular markers related to pancreatic exocrine function to predict the development of PC.

In this study, we found that T-C6 highly expressed KRT13 and FABP5 and clustered in the center of the cancer nest. Previous studies showed that KRT19, KRT17, and KRT7 are highly expressed in PDAC cells (5). We found that these three genes and KRT13 were highly expressed in T-C6 and tumor tissue. However, in tumor tissue, only KRT13 could identify T-C6 from the other seven clusters, suggesting that KRT13 is a specific marker of T-C6. FABP5 may play roles in fatty acid uptake, transport, and metabolism. For the molecular mechanism, FABP5 promoted lipid metabolism reprogramming of hepatocellular carcinoma by enhancing hypoxia-inducible factor-1 alpha activity (32). Although the effects of FABP5 on PC are elusive, our results provided evidence that FABP5 was upregulated in a PC cell subgroup, suggesting that FABP5 may be a new therapeutic target for PC. EPCAM as a membrane protein of epithelial cells, was used as a molecular marker for PC. Our study showed that EPCAM was expressed in both pancreatic ductal cells and cancer cells; however, the expression intensity of cancer cells was higher than that of normal ductal cells, which was similar to the results of single cell sequencing (5). Similar to a previous ST study, we observed the high expression of mucin family genes in cancer cells, such as MUC16, MUC4, and MUC5B (20).

Importantly, to the best of our knowledge, this is the first study to reveal that FGG+CRP+ iCAFs replaced islets in TS. Few studies on the production of CRP in fibroblasts have uncovered a pro-inflammatory role in CRP. For example, plasma exosomes can inhibit CRP production in fibroblasts and induce rheumatoid arthritis (33). IL6-activated human periodontal ligament fibroblasts synthesize CRP, which promotes apical inflammation (34). FGG also activated inflammation and promoted tumor growth. For example, in a rat pulmonary fibrosis model, FGG was highly expressed in fibrotic lung tissue (35). In addition, FGG was found to promote hepatocellular carcinoma cell migration and invasion (36) and, thus, has been validated as a prognostic biomarker for prostate cancer and gastric cancer (37, 38). Several studies have shown that pancreatic CAFs play a vital role in the development of immunosuppressive TME. However, the molecular markers of different subtypes of CAFs remain unclear, hindering the understanding of the roles of CAFs subgroups. The results of our study suggest further investigation of the role of FGG+CRP+ iCAFs in the immune microenvironment of PC.

Because PC is a solid spherical tumor characterized by invasive growth, it is generally assumed that the closer to the tumor center, the earlier the event occurred, and the closer to the tumor edge, the later the event occurred. Thus, the differences in spatial gene expression patterns suggest a dynamic transition between the tumor and the NP. For example, this study found that in TS (tumor center), CAFs primarily possess immunosuppressive activity, whereas in ATT (tumor margin), fibroblasts served an immune-enhancing role. This highlights the results of TME reprogramming. In the future, we should focus on the tumor margin, explore the crosstalk between malignant cells and fibroblasts, and investigate the subsequent remodeling of fibroblasts. In this study, ST revealed that THBS1 levels were higher in fibroblast-enriched tissues than in tumor tissues and NP tissues. It has been reported that exosomal THBS1 promotes stiffness-dependent cancer invasion by engaging matrix metalloproteinases and focal adhesion kinases (39). However, the function of THBS1 in CAFs in PC is poorly understood in PC. At the very least, our results will guide us to explore the effect of fibroblast THBS1 alterations on TME.

The resolution of ST is not as high as that of single-cell transcriptomics, and, therefore, we could not directly determine in which cell a particular gene is expressed. Nevertheless, the spatial gene location, combined with the typical pathological structure of H&E, made it possible to determine in which cell type the gene was expressed. This is a unique advantage of ST in this respect. For example, CD81, a universal cell surface protein, has been increasingly reported as an exosomal biomarker that plays a vital role in PC progression (40). However, it remains challenging to identify the cell source of CD81 protein in a normal or pathological state, even using single-cell sequencing techniques. In this study, ST results indicated that CD81 was primarily distributed in the exocrine part of the normal pancreas and in iCAFs of tumor stroma, showing an increasing trend.

To date, ST still has several limitations. First, the resolution of ST cannot achieve a single-cell level. Each spot actually covers a mixture of dozens of cells; therefore, each spot detects the average value of genes in this small cluster of cells. Second, ST barely recognizes gene expression in rare cells owing to its less precise resolution. For example, CD4 and CD8A (a cell surface glycoprotein in CD8+ T cells) had low and diffuse expression throughout the section. Consequently, we did not evaluate the heterogeneity of immune cells. Third, what we call ST actually refers to transcriptomics in two-dimensional space; however, it cannot describe cell distribution, movement, and polarity in a three-dimensional space scale. After all, ST provided unprecedented insights into the tumor milieu of fewer than 100 μm, which will help us discover new phenomena, mechanisms, and roles in the tumor microenvironment.

In conclusion, we found that during PC progression, the exocrine is more vulnerable than the endocrine; FGG+CRP+ iCAFs displace the islets in the tumor stroma; the fibroblasts at the tumor edge showed immunoenhancement behaviors. Thus, ST enables us to dynamically understand the heterogeneity of the PC microenvironment and inspires us to focus on the predictive role of pancreatic exocrine impairments in PC development.

## Data availability statement

The original contributions presented in the study are publicly available. This data can be found here: GEO database https://www.ncbi.nlm.nih.gov/geo/, GSE327056.

## Ethics statement

The studies involving human participants were reviewed and approved by the Ethics Committee of Beijing Chaoyang Hospital, Capital Medical University. The patients or next of kin provided written informed consent to participate in this study.

## Author contributions

ZR, BP and XZ conceived and designed this study; ZR and BP wrote the manuscript; JZ and XH performed pathological examinations; ZR and SL analyzed the data; FW and ZL collected the samples and conducted the experiments; RL revised the paper; and QH offered administrative support. All authors have read and approved the final manuscript.
