# Correction: Spatial transcriptomics reveals the heterogeneity and FGG+CRP+ inflammatory cancer-associated fibroblasts replace islets in pancreatic ductal adenocarcinoma

**DOI:** 10.3389/fonc.2026.1852367

**Published:** 2026-04-22

**Authors:** Zhangyong Ren, Bing Pan, Fangfei Wang, Shaocheng Lyu, Jialei Zhai, Xiumei Hu, Zhe Liu, Lixin Li, Ren Lang, Qiang He, Xin Zhao

**Affiliations:** 1 Department of Hepatobiliary Surgery, Beijing Chaoyang Hospital affiliated to Capital Medical University, Beijing, China; 2 Department of Pathology, Beijing Chaoyang Hospital affiliated to Capital Medical University, Beijing, China

**Keywords:** pancreatic ductal adenocarcinoma, spatial transcriptomics, tumor microenvironment, heterogeneity, cancer-associated fibroblasts

The **Data availability statement** was erroneously given as “The datasets presented in this study can be found in online repositories. The names of the repository/repositories and accession number(s) can be found in the article/Supplementary Material.”. The correct **Data availability statement** is “The original contributions presented in the study are publicly available. This data can be found here: GEO database https://www.ncbi.nlm.nih.gov/geo/, GSE327056.”.

The original version of this article has been updated.

