# Supplementary material for: Spatial transcriptomics reveals the heterogeneity and FGG+CRP+ inflammatory cancer-associated fibroblasts replace islets in pancreatic ductal adenocarcinoma
Source: Front Oncol. 2023 Apr 14;13:1112576. doi: 10.3389/fonc.2023.1112576 (PMC10140349; doi:10.3389/fonc.2023.1112576)
Supplement: Supplementary file 1 [file DataSheet1.docx]

**Supplementary figures**

Supplementary Figure 1. The Unique Molecular Identifiers (UMI) projection of pathological sections and t-SNE across normal pancreas tissue (A) and pancreatic ductal adenocarcinoma tissues including adjacent tumor tissue (ATT, B), tumor (T, C), and tumor stroma (TS, D). The red color of each spot indicates the UMI count. The darker the spot is, the larger the UMI count is. The bar plot indicates the UMI count.

Supplementary Figure 2. Gene set enrichment analysis based on differential expression genes between pancreatic cancer cells and healthy ductal cells. A. Canonical pathway enrichment reveals that upregulated genes were enriched in “keratinization” and “glycolysis and gluconeogenesis” pathways. B. Downregulated genes were associated with “activation of matrix metalloproteinases” and “EGFR tyrosine inhibitor resistance” pathways.


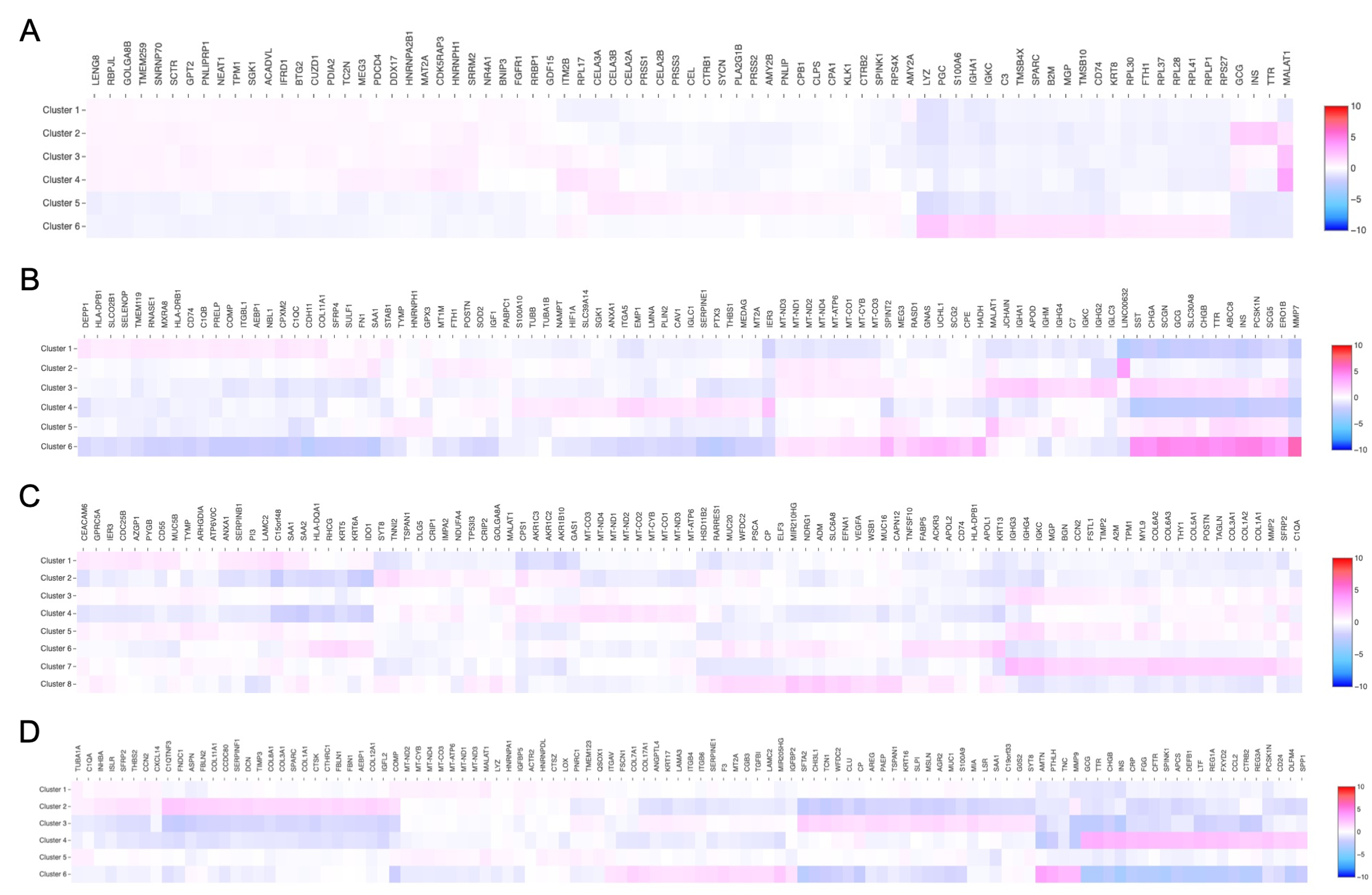


Supplementary Figure 3. Heatmap of top 10 genes of each cluster from normal pancreas (A), adjacent tumor tissue (B), tumor (C), and tumor stroma (D). The bar plot indicates the log_2_FC of the gene expression value of each cluster versus the average gene value.

Supplementary Figure 4. The biological process of adjacent tumor tissue- cluster 1.


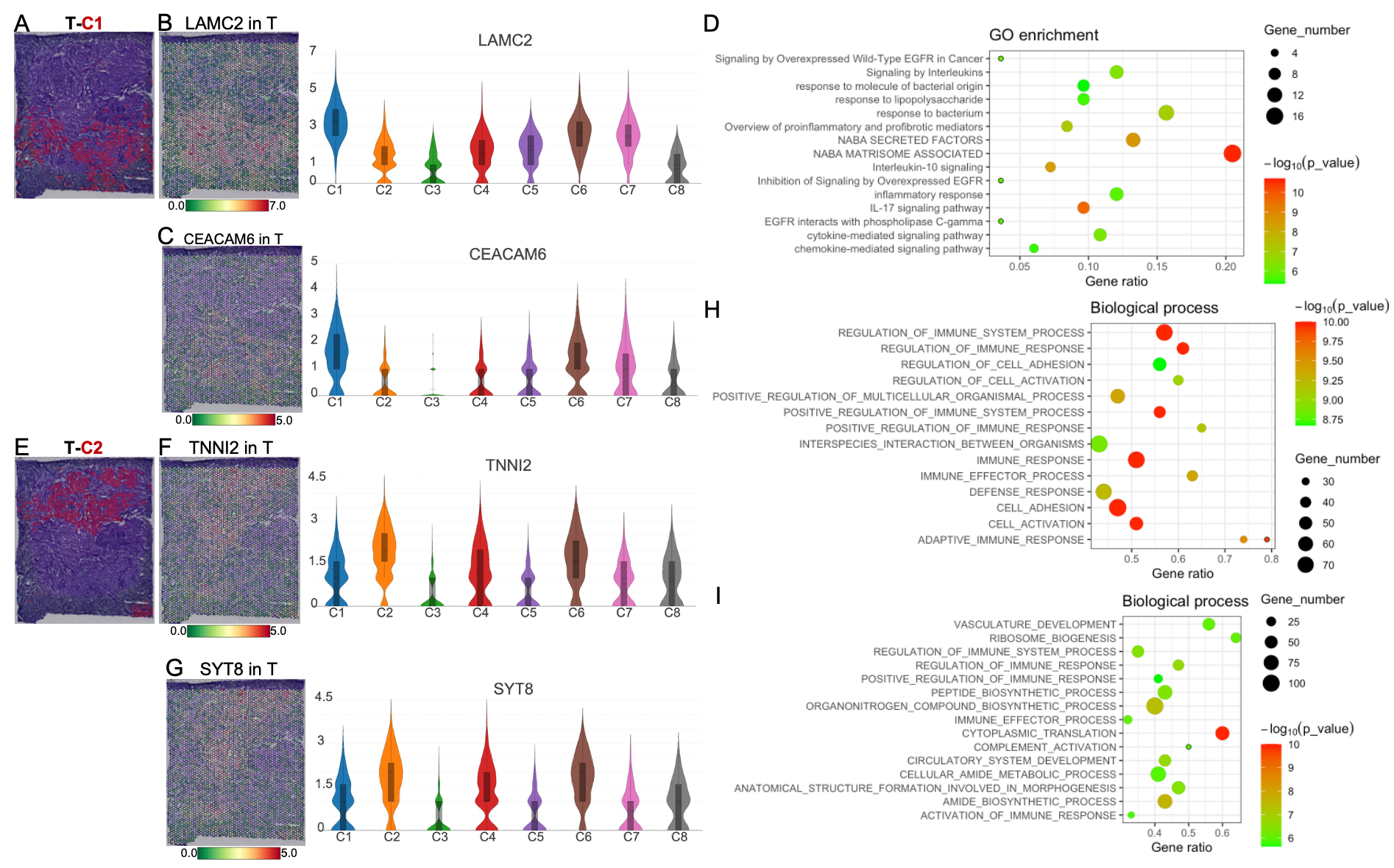


Supplementary Figure 5. Distribution and functions of pancreatic tumor tissue (T)-cluster (C)1 and C2. A. Hematoxylin and eosin (H&E) image shows the distribution of T-C1 in T. The level and distribution of LAMC2 (B) and CEACAM6 (C) in T. D. Biological process of gene set enrichment analysis (GSEA) for T-C1. E. H&E image shows the distribution of T-C2 in T. The level and distribution of TNNI2 (F) and SYT8 (G) in T. H. Biological process of GSEA for T-C2. I. The biological process of GSEA for T-C5.


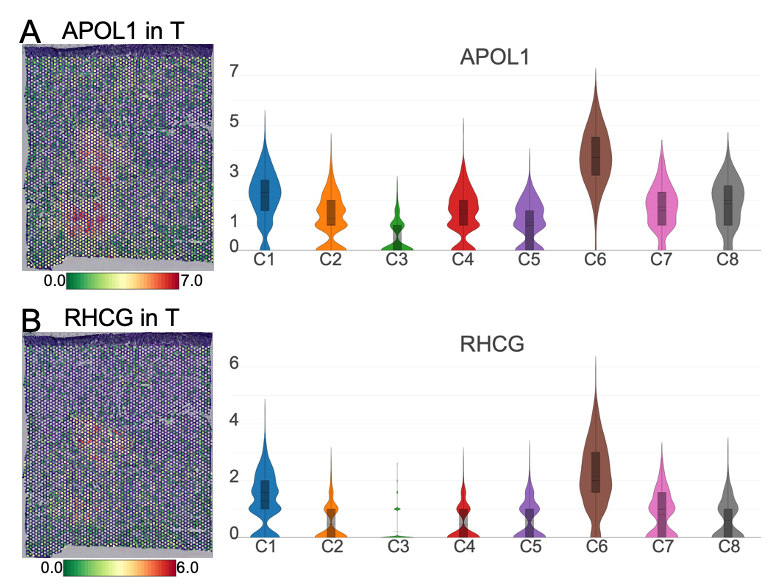


Supplementary Figure 6. The level and distribution of APOL1 (A) and RHCG (B) in pancreatic tumor tissue (T). The bar plots and the vertical coordinate of the violin plots indicates the log2FC of the gene expression value of each cluster versus the average gene value.


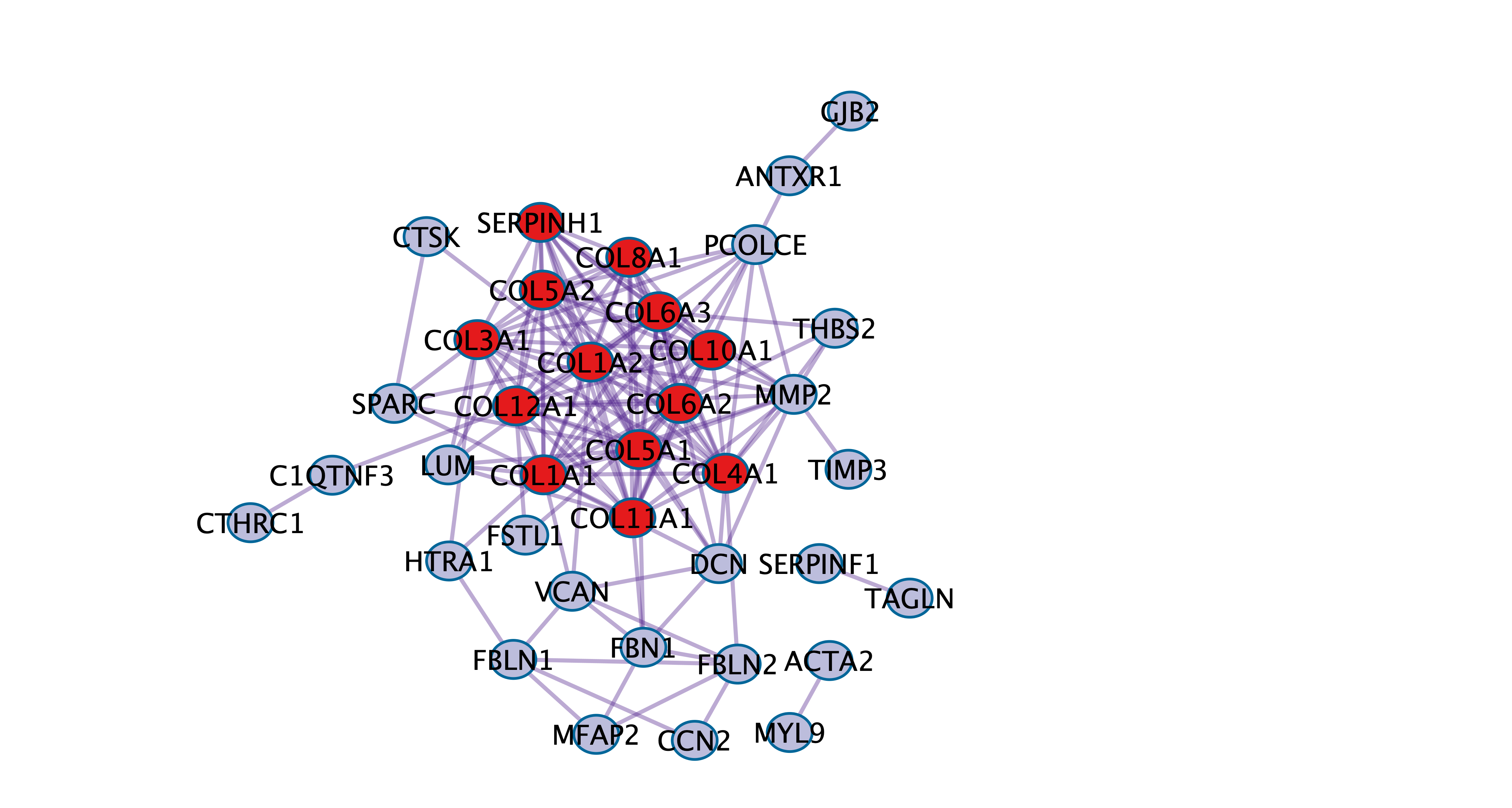


Supplementary Figure 7. Protein-protein interaction network of pancreatic tumor stroma (TS)-cluster 2. The red circles indicate the hub genes of TS-cluster 2.

Supplementary Figure 8. Gene set enrichment analysis (GSEA) of tumor stroma (TS)-cluster (C)1, C5, and C3. A. Canonical pathway enrichment of TS-C1. B and C. The biological process of GSEA for TS-C5 and TS-C3.
