# Supplementary material for: Spatial transcriptomics reveals the heterogeneity and FGG+CRP+ inflammatory cancer-associated fibroblasts replace islets in pancreatic ductal adenocarcinoma
Source: Front Oncol. 2023 Apr 14;13:1112576. doi: 10.3389/fonc.2023.1112576 (PMC10140349; doi:10.3389/fonc.2023.1112576)
Supplement: Supplementary file 2 [file Table1.docx]

Supplementary Table 1. Top ten gene markers for identifying six clusters in normal pancreas.

| Gene | Log2 Fold Change | *p* value | Clusters |
| --- | --- | --- | --- |
| AMY2A | 0.4 | 0.003 | NP-C1 |
| IFRD1 | 0.4 | 0.018 | NP-C1 |
| GOLGA8B | 0.4 | 0.016 | NP-C1 |
| LENG8 | 0.4 | 0.020 | NP-C1 |
| NEAT1 | 0.4 | 0.029 | NP-C1 |
| TC2N | 0.4 | 0.055 | NP-C1 |
| RBPJL | 0.4 | 0.042 | NP-C1 |
| MEG3 | 0.4 | 0.059 | NP-C1 |
| SGK1 | 0.3 | 0.074 | NP-C1 |
| ACADVL | 0.3 | 0.068 | NP-C1 |
| TTR | 2.0 | 0.000 | NP-C2 |
| GCG | 1.9 | 0.000 | NP-C2 |
| INS | 1.9 | 0.000 | NP-C2 |
| MALAT1 | 1.1 | 0.000 | NP-C2 |
| SRRM2 | 0.6 | 0.056 | NP-C2 |
| SCTR | 0.5 | 0.174 | NP-C2 |
| FGFR1 | 0.5 | 0.254 | NP-C2 |
| SNRNP70 | 0.4 | 0.278 | NP-C2 |
| LENG8 | 0.4 | 0.281 | NP-C2 |
| RBPJL | 0.4 | 0.264 | NP-C2 |
| MALAT1 | 2.5 | 0.000 | NP-C3 |
| SRRM2 | 0.6 | 1.000 | NP-C3 |
| HNRNPH1 | 0.6 | 1.000 | NP-C3 |
| ITM2B | 0.6 | 1.000 | NP-C3 |
| GPT2 | 0.6 | 1.000 | NP-C3 |
| LENG8 | 0.5 | 1.000 | NP-C3 |
| CDK5RAP3 | 0.5 | 1.000 | NP-C3 |
| GCG | 0.5 | 1.000 | NP-C3 |
| TMEM259 | 0.5 | 1.000 | NP-C3 |
| RRBP1 | 0.5 | 1.000 | NP-C3 |
| MALAT1 | 3.5 | 0.000 | NP-C4 |
| ITM2B | 1.1 | 1.000 | NP-C4 |
| RPL17 | 1.0 | 1.000 | NP-C4 |
| CDK5RAP3 | 0.9 | 1.000 | NP-C4 |
| HNRNPH1 | 0.8 | 1.000 | NP-C4 |
| GCG | 0.8 | 1.000 | NP-C4 |
| CELA3B | 0.7 | 1.000 | NP-C4 |
| CELA3A | 0.7 | 1.000 | NP-C4 |
| MAT2A | 0.6 | 1.000 | NP-C4 |
| MEG3 | 0.6 | 1.000 | NP-C4 |
| CELA3B | 1.0 | 0.000 | NP-C5 |
| CELA2A | 0.9 | 0.000 | NP-C5 |
| CELA3A | 0.9 | 0.000 | NP-C5 |
| CELA2B | 0.7 | 0.000 | NP-C5 |
| PRSS1 | 0.7 | 0.000 | NP-C5 |
| PNLIP | 0.6 | 0.000 | NP-C5 |
| PRSS3 | 0.6 | 0.001 | NP-C5 |
| PLA2G1B | 0.6 | 0.001 | NP-C5 |
| AMY2B | 0.5 | 0.003 | NP-C5 |
| CLPS | 0.5 | 0.003 | NP-C5 |
| LYZ | 2.2 | 0.000 | NP-C6 |
| PGC | 2.1 | 0.000 | NP-C6 |
| IGKC | 1.7 | 0.000 | NP-C6 |
| IGHA1 | 1.6 | 0.000 | NP-C6 |
| S100A6 | 1.4 | 0.000 | NP-C6 |
| CD74 | 1.2 | 0.000 | NP-C6 |
| B2M | 1.1 | 0.000 | NP-C6 |
| SPARC | 1.1 | 0.000 | NP-C6 |
| C3 | 1.1 | 0.000 | NP-C6 |
| MGP | 1.1 | 0.000 | NP-C6 |
