# Supplementary material for: Spatial transcriptomics reveals the heterogeneity and FGG+CRP+ inflammatory cancer-associated fibroblasts replace islets in pancreatic ductal adenocarcinoma
Source: Front Oncol. 2023 Apr 14;13:1112576. doi: 10.3389/fonc.2023.1112576 (PMC10140349; doi:10.3389/fonc.2023.1112576)
Supplement: Supplementary file 3 [file Table2.docx]

Supplementary Table 2. Top ten gene markers for identifying six clusters in adjacent tumor tissue.

| Gene | Log2 Fold Change | *p* value | Clusters |
| --- | --- | --- | --- |
| CPXM2 | 1.1 | 0.000 | ATT-C1 |
| DEPP1 | 1.0 | 0.000 | ATT-C1 |
| COL11A1 | 1.0 | 0.000 | ATT-C1 |
| TMEM119 | 0.9 | 0.000 | ATT-C1 |
| PRELP | 0.9 | 0.000 | ATT-C1 |
| RNASE1 | 0.9 | 0.000 | ATT-C1 |
| MXRA8 | 0.9 | 0.000 | ATT-C1 |
| ITGBL1 | 0.9 | 0.000 | ATT-C1 |
| C1QC | 0.9 | 0.000 | ATT-C1 |
| STAB1 | 0.8 | 0.000 | ATT-C1 |
| LINC00632 | 0.0 | 0.000 | ATT-C2 |
| SAA1 | 0.1 | 0.084 | ATT-C2 |
| MT-ND3 | 0.4 | 0.391 | ATT-C2 |
| MT-ATP6 | 0.4 | 0.442 | ATT-C2 |
| MT-ND2 | 0.5 | 0.511 | ATT-C2 |
| MT1M | 0.6 | 0.613 | ATT-C2 |
| POSTN | 0.8 | 0.764 | ATT-C2 |
| MALAT1 | 1.0 | 1.000 | ATT-C2 |
| MT-ND1 | 1.0 | 1.000 | ATT-C2 |
| SOD2 | 1.0 | 1.000 | ATT-C2 |
| APOD | 2.1 | 0.000 | ATT-C3 |
| IGHA1 | 1.9 | 0.000 | ATT-C3 |
| JCHAIN | 1.9 | 0.000 | ATT-C3 |
| IGHG2 | 1.8 | 0.000 | ATT-C3 |
| SST | 1.8 | 0.000 | ATT-C3 |
| CHGA | 1.7 | 0.000 | ATT-C3 |
| MALAT1 | 1.6 | 0.000 | ATT-C3 |
| IGLC3 | 1.6 | 0.000 | ATT-C3 |
| SLC30A8 | 1.5 | 0.000 | ATT-C3 |
| IGHM | 1.5 | 0.000 | ATT-C3 |
| IER3 | 2.4 | 0.000 | ATT-C4 |
| EMP1 | 1.6 | 0.000 | ATT-C4 |
| ITGA5 | 1.4 | 0.000 | ATT-C4 |
| SERPINE1 | 1.2 | 0.000 | ATT-C4 |
| LMNA | 1.2 | 0.000 | ATT-C4 |
| PTX3 | 1.2 | 0.000 | ATT-C4 |
| CAV1 | 1.1 | 0.000 | ATT-C4 |
| PLIN2 | 1.1 | 0.000 | ATT-C4 |
| HIF1A | 1.0 | 0.000 | ATT-C4 |
| MEDAG | 0.9 | 0.000 | ATT-C4 |
| MALAT1 | 2.3 | 1.000 | ATT-C5 |
| ABCC8 | 1.8 | 1.000 | ATT-C5 |
| TTR | 1.5 | 1.000 | ATT-C5 |
| INS | 1.4 | 1.000 | ATT-C5 |
| SPINT2 | 1.2 | 1.000 | ATT-C5 |
| SCG5 | 1.1 | 1.000 | ATT-C5 |
| IGHG4 | 1.1 | 1.000 | ATT-C5 |
| MEG3 | 1.0 | 1.000 | ATT-C5 |
| CHGA | 1.0 | 1.000 | ATT-C5 |
| PCSK1N | 0.9 | 1.000 | ATT-C5 |
| MMP7 | 6.5 | 0.000 | ATT-C6 |
| INS | 4.9 | 0.000 | ATT-C6 |
| SCGN | 4.9 | 0.000 | ATT-C6 |
| PCSK1N | 4.9 | 0.000 | ATT-C6 |
| CHGB | 4.7 | 0.000 | ATT-C6 |
| GCG | 4.6 | 0.000 | ATT-C6 |
| ABCC8 | 4.6 | 0.000 | ATT-C6 |
| SLC30A8 | 4.5 | 0.000 | ATT-C6 |
| G6PC2 | 4.5 | 0.000 | ATT-C6 |
| SST | 4.4 | 0.000 | ATT-C6 |
