# Supplementary material for: Spatial transcriptomics reveals the heterogeneity and FGG+CRP+ inflammatory cancer-associated fibroblasts replace islets in pancreatic ductal adenocarcinoma
Source: Front Oncol. 2023 Apr 14;13:1112576. doi: 10.3389/fonc.2023.1112576 (PMC10140349; doi:10.3389/fonc.2023.1112576)
Supplement: Supplementary file 4 [file Table3.docx]

Supplementary Table 3. Top ten gene markers for identifying eight clusters in tumor tissue.

| Gene | Log2 Fold Change | *p* value | Clusters |
| --- | --- | --- | --- |
| C15orf48 | 1.4 | 0.000 | T-C1 |
| LAMC2 | 1.1 | 0.000 | T-C1 |
| AZGP1 | 1.1 | 0.000 | T-C1 |
| PI3 | 0.9 | 0.000 | T-C1 |
| CEACAM6 | 0.9 | 0.000 | T-C1 |
| CDC25B | 0.9 | 0.000 | T-C1 |
| CD55 | 0.9 | 0.000 | T-C1 |
| SERPINB1 | 0.9 | 0.000 | T-C1 |
| MUC5B | 0.9 | 0.000 | T-C1 |
| ANXA1 | 0.8 | 0.000 | T-C1 |
| TNNI2 | 1.4 | 0.000 | T-C2 |
| SYT8 | 1.2 | 0.000 | T-C2 |
| CPS1 | 1.2 | 0.000 | T-C2 |
| TSPAN1 | 0.8 | 0.007 | T-C2 |
| IMPA2 | 0.8 | 0.023 | T-C2 |
| GAS1 | 0.7 | 0.040 | T-C2 |
| DLG5 | 0.7 | 0.046 | T-C2 |
| HSD11B2 | 0.7 | 0.052 | T-C2 |
| CRIP1 | 0.7 | 0.075 | T-C2 |
| PSCA | 0.7 | 0.112 | T-C2 |
| IGHG4 | 1.5 | 1.000 | T-C3 |
| IGHG3 | 1.3 | 1.000 | T-C3 |
| IGKC | 1.2 | 1.000 | T-C3 |
| MALAT1 | 0.8 | 1.000 | T-C3 |
| CCN2 | 0.7 | 1.000 | T-C3 |
| MGP | 0.7 | 1.000 | T-C3 |
| MMP2 | 0.7 | 1.000 | T-C3 |
| BGN | 0.7 | 1.000 | T-C3 |
| GOLGA8A | 0.7 | 1.000 | T-C3 |
| MYL9 | 0.7 | 1.000 | T-C3 |
| MT-ND2 | 1.3 | 0.000 | T-C4 |
| MT-ND4 | 1.2 | 0.000 | T-C4 |
| MT-CO3 | 1.2 | 0.000 | T-C4 |
| MT-CYB | 1.1 | 0.000 | T-C4 |
| SFRP2 | 1.1 | 0.000 | T-C4 |
| MT-ND1 | 1.1 | 0.000 | T-C4 |
| CPS1 | 1.0 | 0.000 | T-C4 |
| MT-ND3 | 1.0 | 0.000 | T-C4 |
| GAS1 | 0.9 | 0.002 | T-C4 |
| AKR1B10 | 0.9 | 0.002 | T-C4 |
| IGHG4 | 1.5 | 0.100 | T-C5 |
| IGKC | 1.4 | 0.903 | T-C5 |
| IGHG3 | 1.2 | 0.909 | T-C5 |
| MYL9 | 1.0 | 0.903 | T-C5 |
| TYMP | 0.9 | 1.000 | T-C5 |
| C1QA | 0.9 | 1.000 | T-C5 |
| MMP2 | 0.8 | 1.000 | T-C5 |
| CCN2 | 0.7 | 1.000 | T-C5 |
| C15orf48 | 0.7 | 1.000 | T-C5 |
| ATP6V0C | 0.7 | 1.000 | T-C5 |
| KRT13 | 2.2 | 0.000 | T-C6 |
| APOL1 | 1.5 | 0.000 | T-C6 |
| KRT5 | 1.5 | 0.000 | T-C6 |
| RHCG | 1.4 | 0.000 | T-C6 |
| FABP5 | 1.3 | 0.000 | T-C6 |
| TNFSF10 | 1.2 | 0.000 | T-C6 |
| HLA-DQA1 | 1.2 | 0.000 | T-C6 |
| CD74 | 1.1 | 0.000 | T-C6 |
| KRT6A | 1.1 | 0.000 | T-C6 |
| MUC20 | 1.1 | 0.000 | T-C6 |
| IGHG3 | 2.7 | 0.000 | T-C7 |
| IGKC | 2.3 | 0.000 | T-C7 |
| IGHG4 | 2.3 | 0.000 | T-C7 |
| COL6A2 | 1.9 | 0.000 | T-C7 |
| MMP2 | 1.9 | 0.000 | T-C7 |
| COL6A3 | 1.8 | 0.000 | T-C7 |
| TAGLN | 1.8 | 0.000 | T-C7 |
| THY1 | 1.8 | 0.000 | T-C7 |
| POSTN | 1.8 | 0.000 | T-C7 |
| COL1A1 | 1.7 | 0.000 | T-C7 |
| MIR210HG | 2.2 | 0.025 | T-C8 |
| ADM | 2.1 | 0.015 | T-C8 |
| EFNA1 | 1.8 | 0.093 | T-C8 |
| NDRG1 | 1.8 | 0.117 | T-C8 |
| SLC6A8 | 1.7 | 0.278 | T-C8 |
| CAPN12 | 1.6 | 0.526 | T-C8 |
| MUC16 | 1.6 | 0.415 | T-C8 |
| MUC20 | 1.6 | 0.632 | T-C8 |
| WFDC2 | 1.4 | 0.933 | T-C8 |
| PSCA | 1.4 | 0.859 | T-C8 |
