# Supplementary material for: Spatial transcriptomics reveals the heterogeneity and FGG+CRP+ inflammatory cancer-associated fibroblasts replace islets in pancreatic ductal adenocarcinoma
Source: Front Oncol. 2023 Apr 14;13:1112576. doi: 10.3389/fonc.2023.1112576 (PMC10140349; doi:10.3389/fonc.2023.1112576)
Supplement: Supplementary file 5 [file Table4.docx]

Supplementary Table 4. Top ten gene markers for identifying six clusters in tumor stroma tissue.

| Gene | Log2 Fold Change | *p* value | Clusters |
| --- | --- | --- | --- |
| MALAT1 | 0.7 | 0.044 | TS-C1 |
| TUBA1A | 0.6 | 0.193 | TS-C1 |
| COMP | 0.6 | 0.287 | TS-C1 |
| CCN2 | 0.6 | 0.271 | TS-C1 |
| MT-ND4 | 0.6 | 0.284 | TS-C1 |
| MT-ATP6 | 0.5 | 0.412 | TS-C1 |
| MT-ND2 | 0.5 | 0.591 | TS-C1 |
| MT-CYB | 0.5 | 0.591 | TS-C1 |
| MT-CO3 | 0.5 | 0.591 | TS-C1 |
| CXCL14 | 0.5 | 0.682 | TS-C1 |
| C1QTNF3 | 2.2 | 0.000 | TS-C2 |
| FNDC1 | 2.2 | 0.000 | TS-C2 |
| ASPN | 1.8 | 0.000 | TS-C2 |
| FBLN2 | 1.8 | 0.000 | TS-C2 |
| COL11A1 | 1.8 | 0.000 | TS-C2 |
| COL12A1 | 1.8 | 0.000 | TS-C2 |
| DCN | 1.7 | 0.000 | TS-C2 |
| CTSK | 1.7 | 0.000 | TS-C2 |
| CCDC80 | 1.6 | 0.000 | TS-C2 |
| FBLN1 | 1.6 | 0.000 | TS-C2 |
| SFTA2 | 1.9 | 0.000 | TS-C3 |
| AGR2 | 1.7 | 0.000 | TS-C3 |
| TCN1 | 1.7 | 0.000 | TS-C3 |
| MIA | 1.6 | 0.000 | TS-C3 |
| PAEP | 1.6 | 0.000 | TS-C3 |
| AREG | 1.6 | 0.000 | TS-C3 |
| CHI3L1 | 1.6 | 0.000 | TS-C3 |
| SLPI | 1.6 | 0.000 | TS-C3 |
| MSLN | 1.5 | 0.000 | TS-C3 |
| WFDC2 | 1.4 | 0.000 | TS-C3 |
| TTR | 3.2 | 0.000 | TS-C4 |
| GCG | 3.1 | 0.000 | TS-C4 |
| INS | 2.9 | 0.000 | TS-C4 |
| CHGB | 2.8 | 0.000 | TS-C4 |
| FGG | 2.7 | 0.000 | TS-C4 |
| CRP | 2.6 | 0.000 | TS-C4 |
| LTF | 2.6 | 0.000 | TS-C4 |
| CFTR | 2.6 | 0.000 | TS-C4 |
| SPINK1 | 2.6 | 0.000 | TS-C4 |
| APCS | 2.5 | 0.000 | TS-C4 |
| MALAT1 | 0.8 | 1.000 | TS-C5 |
| PNRC1 | 0.7 | 1.000 | TS-C5 |
| TUBA1A | 0.6 | 1.000 | TS-C5 |
| C1QA | 0.6 | 1.000 | TS-C5 |
| HNRNPDL | 0.6 | 1.000 | TS-C5 |
| MT-ND4 | 0.6 | 1.000 | TS-C5 |
| MT-CYB | 0.6 | 1.000 | TS-C5 |
| QSOX1 | 0.6 | 1.000 | TS-C5 |
| MT-ND2 | 0.5 | 1.000 | TS-C5 |
| LOX | 0.5 | 1.000 | TS-C5 |
| AMTN | 3.6 | 0.000 | TS-C6 |
| PTHLH | 3.2 | 0.000 | TS-C6 |
| TNC | 2.7 | 0.000 | TS-C6 |
| MMP9 | 2.7 | 0.000 | TS-C6 |
| MIR205HG | 2.1 | 0.000 | TS-C6 |
| COL7A1 | 1.8 | 0.000 | TS-C6 |
| F3 | 1.7 | 0.000 | TS-C6 |
| CGB3 | 1.5 | 0.000 | TS-C6 |
| COL17A1 | 1.5 | 0.000 | TS-C6 |
| SERPINE1 | 1.4 | 0.000 | TS-C6 |
